# Modulator approach for the design and synthesis of anisotropic multi-domain metal–organic frameworks†

**DOI:** 10.1039/d4sc07985j

**Published:** 2025-03-20

**Authors:** Yiwen He, Zhehao Li, Zoe M. Soilis, Gefan He, Nathaniel L. Rosi

**Affiliations:** a Department of Chemistry, University of Pittsburgh Pittsburgh Pennsylvania 15260 USA nrosi@pitt.edu; b Department of Chemical and Petroleum Engineering, University of Pittsburgh Pittsburgh Pennsylvania 15261 USA

## Abstract

Multi-domain metal–organic frameworks (MD-MOFs) consist of chemically-distinct interconnected MOF domains. Most commonly they are isotropic, with core–shell and stratified MOFs representing classic examples in which a core MOF is concentrically encased in one or more MOF shells. Anisotropic multi-domain MOFs (AMD-MOFs) are much rarer and are projected to exhibit unique properties that depend on domain sequence, composition, and 3-D spacial distribution. However, straightforward approaches for their synthesis and construction are underdeveloped. We present and describe a modulator-based strategy for preparing a diverse collection of AMD-MOFs. Designed coordination modulators were used to inhibit secondary domain growth along certain facets of seed MOF crystals. Through multistep syntheses, this strategy allows for controlled construction of AMD-MOFs with different domain distributions that depend on modulator identity and domain synthesis sequence. The reported results represent important steps toward realizing a more general synthetic approach for fabricating arbitrarily complex AMD-MOFs, which is crucial for enabling broader exploration and study of their properties, functions, and applications.

## Abstract 2

A molecular modulator approach for controlling the placement and growth of MOF domains is reported. This method enables facile creation of anisotropic multi-domain MOFs, expanding the scope of MOF hierarchical structural and functional complexity.

## Graphical abstract

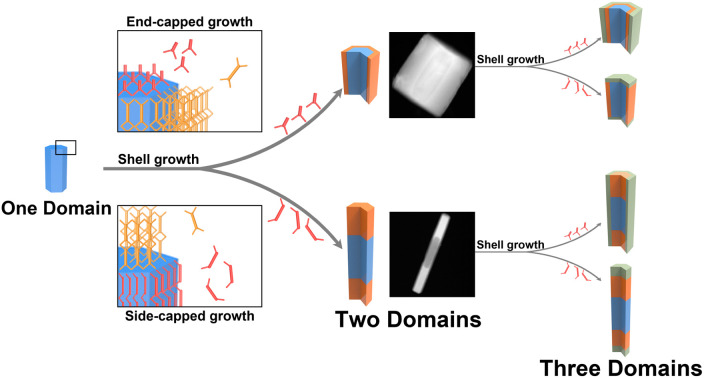

## Introduction

The connectivity and spatial disposition of chemical moieties and different functional domains underscore the properties of all matter. Precise synthetic control over the placement of these constituents allows for the design and rational fabrication of functional molecules and materials, from complex natural products^1,2^ and multiblock polymers^3–6^ to multicomponent and multi-domain nanoparticles^7,8^ and metal–organic frameworks (MOFs).^9–14^

MOFs, in particular, exhibit multiple levels of chemical and structural complexity. At the molecular level, they consist of periodically interconnected metal and organic components (*n*), where *n* ≥ 2, and their properties and functions are largely defined by the identity and 3-D connectivity (*i.e.*, MOF topology) of these components.^15–17^ Strategies for further increasing MOF complexity have emerged which rely on considering MOFs themselves as domain building blocks^18,19^ in larger scale ‘MOF-on-MOF’ architectures.^20–35^ While fabrication methods for such materials have advanced considerably, it remains challenging to rationally construct anisotropic multi-domain MOFs (AMD-MOFs) consisting of multiple different interconnected MOF domains.^29,36–40^

To contextualize this challenge, it is instructive to relate multi-domain MOF syntheses to the derivatization of organic molecular substrates. If the substrate has multiple functional groups with similar reactivity, an undiscriminating reactant may react with all of them. Analogously, if MOF shell growth is equally likely on all facets of a MOF seed crystal, then isotropic shell growth will occur, resulting in the formation of isotropic core–shell MOF products.^20,21,25,41^ Targeting reactions to specific functional groups of an organic substrate can result in selective derivatization; however, this requires careful choice of reactants and synthetic conditions. For comparison, there are rare cases of multi-domain MOF syntheses that result in facet-selective shell growth and the formation of AMD-MOFs, yet they necessitate meticulous selection of MOF seed and shell pairs.^29,36–40^ In organic synthesis, a general strategy to prevent reaction at some functional groups—and thus direct reactions toward others—involves use of protecting groups. A similar widely applicable approach for prescribing secondary MOF domain growth to specific facets of a MOF seed crystal is lacking.

Drawing inspiration from organic protecting group strategies, we envisioned that molecular coordination modulators could be designed to prevent shell growth on specific MOF facets while allowing it to occur on others.^42^ Indeed, introducing molecular modulators to MOF syntheses has proven to be an effective way of controlling MOF crystal growth and influencing crystal morphology.^42–46^ Modulators can affect the pH of the reaction medium or competitively coordinate to metal sites, limiting crystal growth along certain axes. A modulator-based approach could make AMD-MOF fabrication more straightforward and potentially more general than current established methods.

**Fig. 1:**
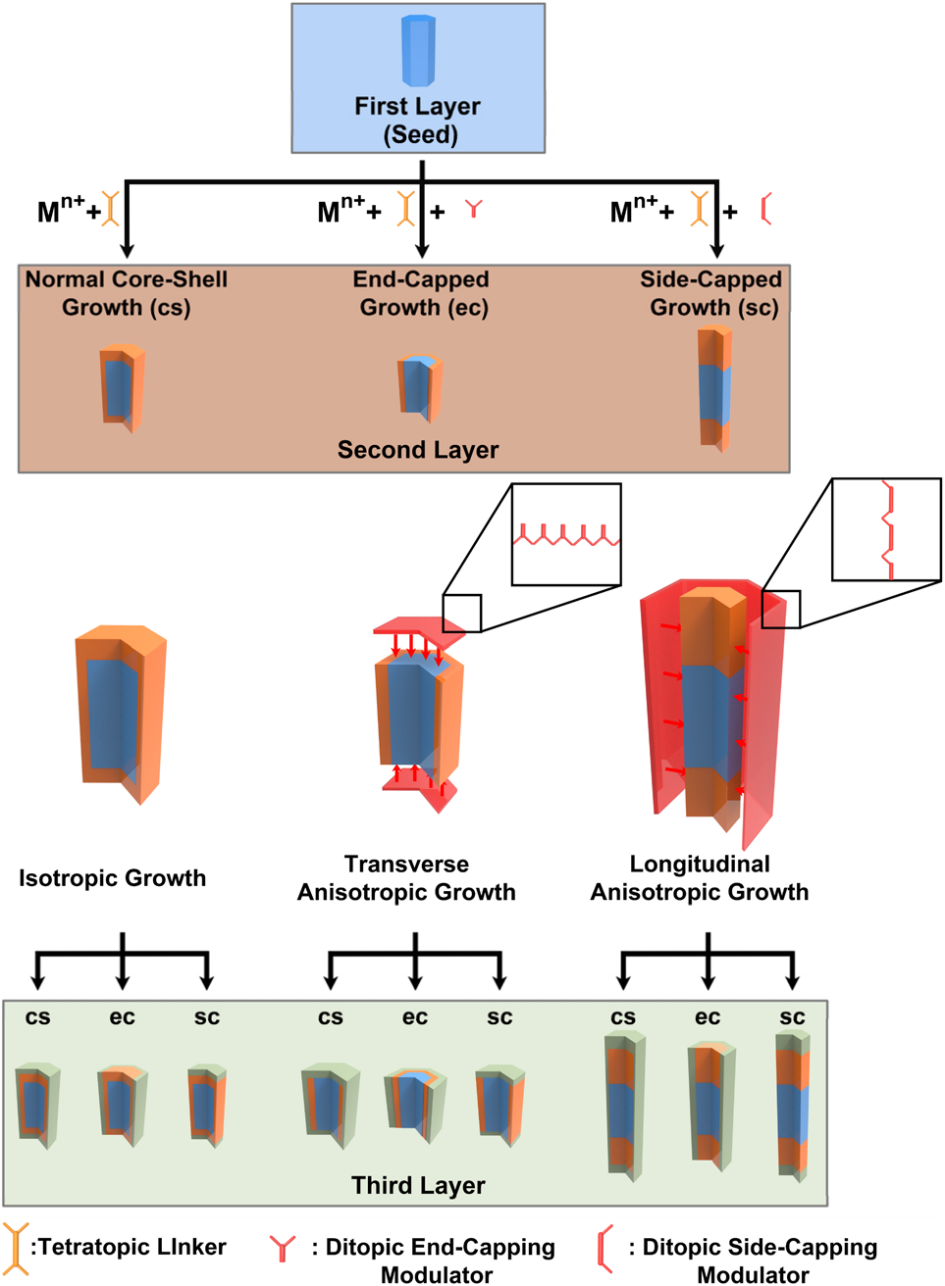
Synthetic strategy for fabricating AMD-MOFs.

Our proposed strategy for controlling domain growth is illustrated in Fig. 1. In a typical synthesis, isotropic shell growth yields a core–shell (cs) MOF (left). To direct either transverse or longitudinal secondary domain growth, a molecular modulator (red) designed to competitively bind to either the seed crystal's ends (end cap, ec) or sides (side cap, sc), respectively, would be added to the synthesis (middle and right). After secondary domain growth, which could result in three potential binary domain MOFs, a third phase of modulated or unmodulated growth would yield a diverse collection of 9 different ternary domain MOFs, with 8 exhibiting anisotropic disposition of domains. Successive domain growth steps could be implemented to generate increasingly complex families of AMD-MOFs.

In this study, we design ditopic modulators to direct the formation and structure of AMD-MOFs. We first synthesize binary domain MOFs and characterize their morphology and composition using a variety of microscopic and spectroscopic methods. After demonstrating modulator-based control over secondary domain growth, we explore the scope of the methodology through design and synthesis of multiple different ternary AMD-MOFs. The results (i) illustrate the utility of a modulator-guided synthetic approach for fabricating diverse collections of AMD-MOFs; (ii) contribute to the fundamental understanding of MOF growth mechanisms; and (iii) provide practical routes for creating multi-MOF systems with tailorable structures and compositions.

## Results and discussion

### Anisotropic growth of binary domain MOFs

Gu *et al.* introduced a linker scissoring strategy to control the synthesis and growth of non-cubic MOF crystals constructed using tetratopic carboxylate linkers.^47^ They designed ditopic modulators derived from the tetratopic linkers to direct either transverse or longitudinal MOF crystal growth, resulting in formation of either nanoplatelets or nanorods. We reasoned a similar strategy could be adopted to positionally direct secondary MOF growth onto MOF seed crystals, thereby facilitating the controlled fabrication of AMD-MOFs.

**Fig. 2:**
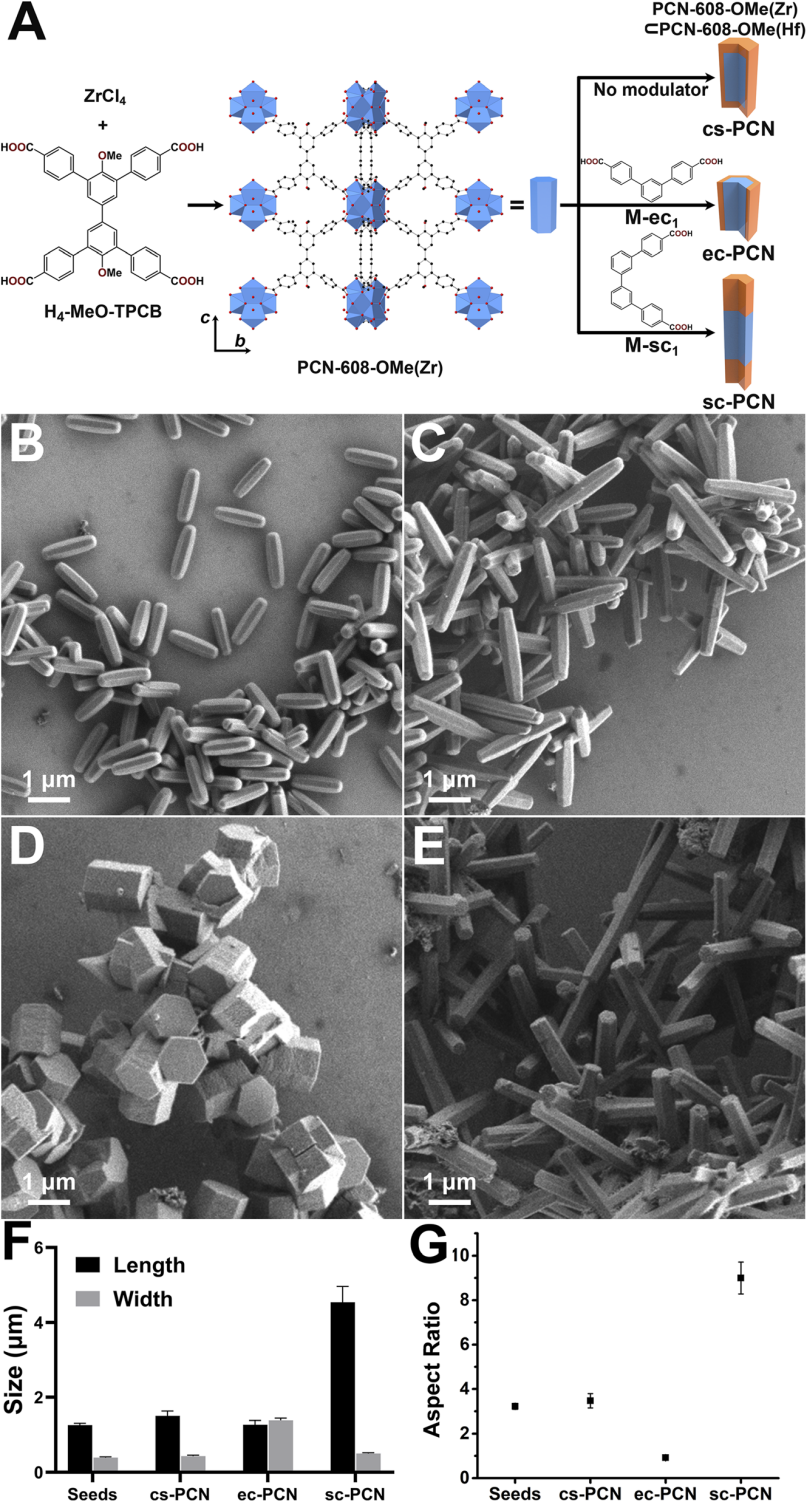
Illustration of binary domain PCN MOF syntheses and crystal structure of PCN-608-OMe(Zr) (Zr^4+^, blue polyhedra; C, black spheres; O, red spheres; H omitted for clarity) (A); SEM images for PCN-608-OMe seeds (B), cs-PCN (C), ec-PCN (D), SC-PCN (E); sizes (F) and aspect ratios (G) of seeds and binary domain PCN MOFs (each point in (F) and (G) is the average of 100 counts and the error bars represent the standard deviation of the mean).

We chose PCN-608-OMe^48^ to investigate the applicability of this approach. PCN-608-OMe consists of Zr_6_O_8_ clusters interlinked by the *D*_2h_-symmetric tetratopic ligand, 4,4′-dimethoxybiphenyl-3,3′,5,5′-tetra(phenyl-4-carboxylate) (MeO-TPCB) (Fig. 2A). Within the PCN-608-OMe crystal structure, the long axis of MeO-TPCB aligns with the *c* crystallographic axis. Rod-like seed crystals of PCN-608-OMe were first synthesized. The average crystal length (1.26 ± 0.05 μm), width (0.39 ± 0.02 μm), and aspect ratio (3.22 ± 0.13) were measured from scanning electron microscopy (SEM) images (100 counts; Fig. 2B, F, G, S18 and Table S2†). Secondary domain growth solutions consisting of HfCl_4_, trifluoroacetic acid (TFA), H_4_-MeO-TPCB and dimethylformamide (DMF) were then prepared; Hf(iv) was used instead of Zr(iv) to distinguish domains using elemental mapping (*vide infra*). The seed crystals were immersed and heated (120 °C, 20 h) in the secondary growth solution yielding PCN-608-OMe(Zr)⊂PCN-608-OMe(Hf), binary ‘core⊂shell’ product hereafter denoted as cs-PCN. Powder X-ray diffraction (PXRD) analyses reveal that both the seed and core⊂shell MOFs are crystalline, phase pure, and isostructural to PCN-608 (Fig. S17 and S23†). cs-PCN crystals were longer (1.50 ± 0.13 μm) and wider (0.43 ± 0.03 μm) than the seed crystals (Fig. 2C, F, S25 and Table S3†), yet their aspect ratio (3.47 ± 0.32) (Fig. 2G and Table S3†) was comparable.

Having established synthetic conditions for preparing well-defined seed crystals and for secondary domain growth, we proceeded to explore syntheses that incorporated modulators to positionally direct growth of secondary domains onto specific seed crystal facets. Guided by the linker scissoring strategy summarized above,^47^ we prepared two different ditopic modulators based on the tetratopic MeO-TPCB linker. (1,1′,3′,1′′-Terphenyl)-4,4′′-dicarboxylic acid was designed to competitively coordinate along (001) and serve as an end-capping modulator (M-ec_1_) for directing transverse secondary domain growth. (1,1′,3′,1'':3′′,1′′′-Quaterphenyl)-4,4′′′-dicarboxylic acid was designed as a side-capping modulator (M-sc_1_) to coordinate along both (100) and (010) and thus direct longitudinal secondary domain growth. PCN-608-OMe seed crystals were immersed and heated in secondary domain growth solutions containing either M-ec_1_ or M-sc_1_ (120 °C, 20 h). These syntheses yielded anisotropic binary domain MOFs denoted as either end-capped PCN-608-OMe(Zr)⊂PCN-608-OMe(Hf) (ec-PCN) or side-capped PCN-608-OMe(Zr)⊂PCN-608- OMe(Hf) (sc-PCN). PXRD patterns revealed that ec-PCN and sc-PCN are isostructural to PCN-608 (Fig. S23†), and SEM was used to determine their size distributions and aspect ratios (Fig. 2D and E). The dimensions of the ec-PCN and sc-PCN were compared to the dimensions of the seed crystals to assess how the modulators influenced shell growth. The length of ec-PCN was determined to be 1.27 ± 0.11 μm (Fig. 2F, S26 and Table S3†), indicating minimal growth along the longitudinal axis (*cf.* 1.26 ± 0.05 μm for seed crystal). In contrast, the length of sc-PCN was 4.54 ± 0.43 μm (Fig. 2F, S27 and Table S3†), approximately 3× longer than the seed crystals. ec-PCN and sc-PCN exhibited widths of 1.38 ± 0.06 μm and 0.50 ± 0.02 μm, respectively (Fig. 2F, S26, S27 and Table S3†), indicating transverse secondary domain growth was significant for ec-PCN and limited for sc-PCN (*cf.* 0.39 ± 0.02 μm for seed crystals). The differences become even more pronounced when comparing the aspect ratios of ec-PCN and sc-PCN to that of the seed crystals. As previously mentioned, the aspect ratios of cs-PCN and the PCN-608-OMe(Zr) seeds are nearly identical. However, both are three times higher than that of ec-PCN (0.92 ± 0.06, Fig. 2G and Table S3†). A similarly large disparity is observed for sc-PCN, which exhibits an aspect ratio of 9.00 ± 0.72 (Fig. 2G and Table S3†), nearly three times greater than the original PCN-608-OMe(Zr) seed. Based on the dimensions and aspect ratios of ec-PCN and sc-PCN, we conclude that addition of M-ec_1_ or M-sc_1_ modulators to the syntheses significantly affects the location and direction of secondary domain growth.

**Fig. 3:**
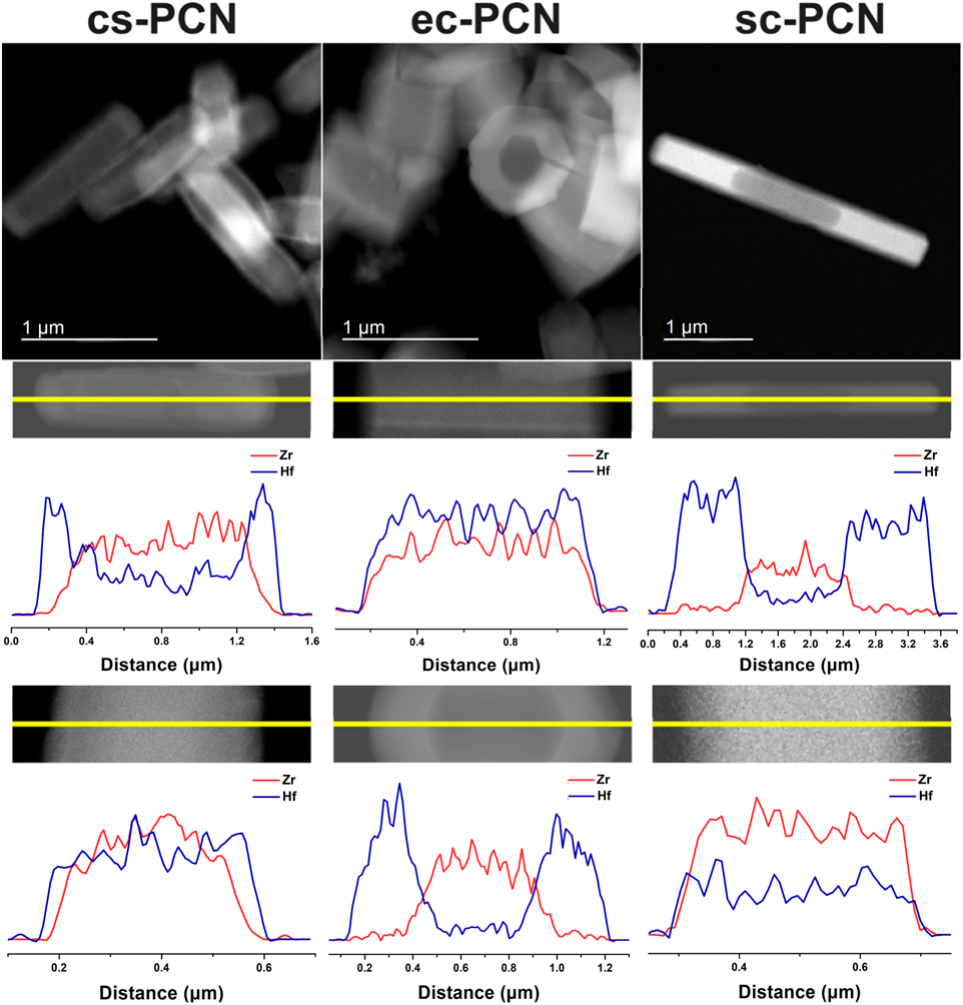
HAADF images for binary domain PCN MOFs (top); STEM-EDS line-scans along longitudinal (middle) and transverse (bottom) directions.

Scanning transmission electron microscopy (STEM) was used to collect high-angle annular dark-field (HAADF) images and energy dispersive X-ray spectroscopy (EDS) line-scans to further elucidate and map MOF domain distribution (Fig. 3). In each case, the outer secondary domain is brighter than the inner seed region due to the presence of Hf. The HAADF image of cs-PCN indicated growth of PCN-608-OMe(Hf) in both the longitudinal and transverse directions. This observation was further corroborated by STEM-EDS line-scans, which revealed Zr signals in the inner region and Hf signals in the outer region of the crystals. During secondary domain growth, both metal and linker exchange is possible, and these processes have been documented in this context in previous publications by us and others.^24,28^ From the data presented, we can reasonably conclude that the secondary domain primarily contains Hf^4+^ and the seed domain is primarily Zr^4+^. As a control experiment, PCN-608-OMe seed crystals were immersed and heated (120 °C, 20 h) in the same secondary growth solution but without the ligand or modulator. HAADF image and STEM-EDS confirm the absence of Hf^4+^ in the resulting MOFs, indicating undetectable metal exchange during secondary growth (Fig. S19†). HAADF images and EDS line-scans of products from the modulated syntheses are starkly different than those for cs-PCN. We observe that ec-PCN almost exclusively exhibits transverse secondary domain growth while secondary domain growth for sc-PCN is almost solely along the longitudinal direction. Collectively, the data indicate that the designed ditopic modulators (i) inhibit secondary domain growth on certain facets; (ii) direct the placement and growth of domains to specific locations; and (iii) enable rational construction of anisotropic binary domain MOFs.

**Fig. 4:**
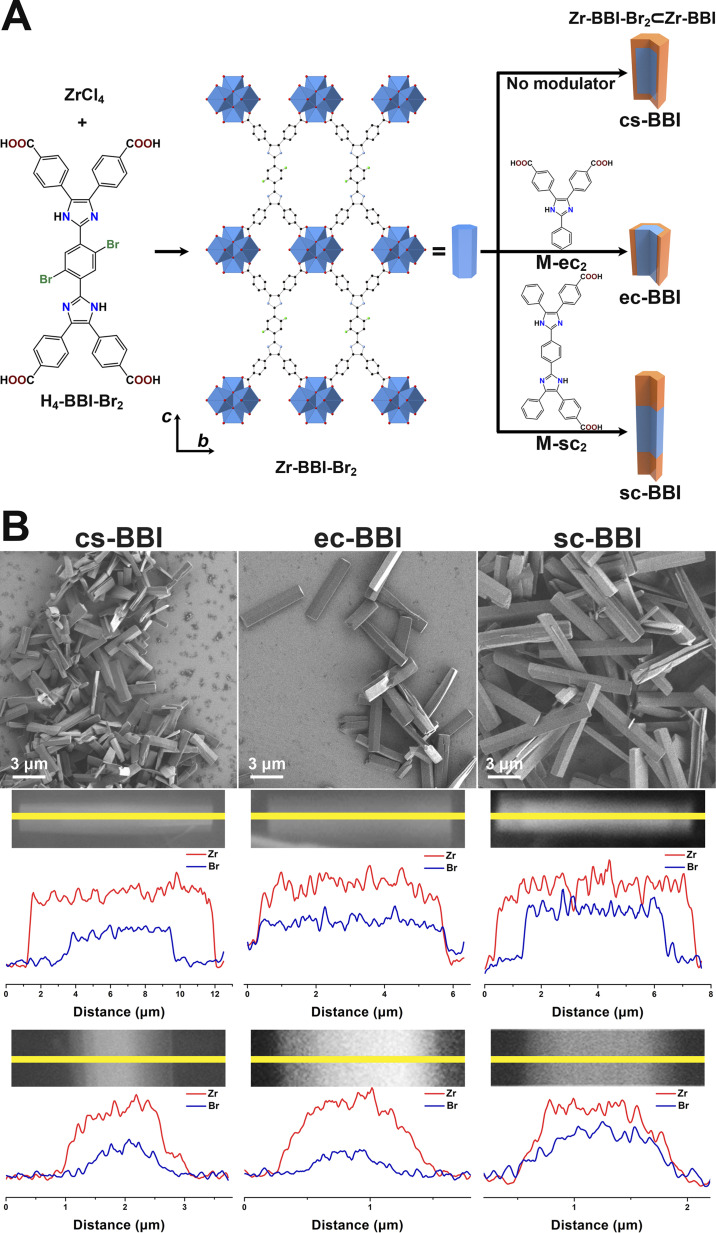
Illustration of binary domain Zr-BBI MOF syntheses and crystal structure of Zr-BBI-Br_2_ (Zr^4+^, blue polyhedra; C, black spheres; O, red spheres; Br, green spheres; H omitted for clarity) (A); SEM images for binary Zr-BBI MOFs (B, top); SEM-EDS line-scans along longitudinal (B, middle) and transverse (B, bottom) directions.

We applied this strategy to a second MOF system, Zr-BBI,^49^ to explore its versatility. Like PCN-608-OMe, Zr-BBI consists of Zr_6_O_8_ cluster nodes interconnected by tetratopic *D*_2h_ symmetric linkers, BBI (4,4′,4′′,4′′′-(1,4-phenylenebis(1*H*-imidazole-2,4,5- triyl))tetrabenzoate). We used a brominated version of BBI (BBI-Br_2_, Fig. 4A), to prepare seed crystals so that we could identify its location using SEM-EDS Br mapping. Secondary domains were grown using non-brominated BBI. Zr-BBI-Br_2_ seeds were immersed and heated in a growth solution (100 °C, 20 h) to synthesize Zr-BBI-Br_2_⊂Zr-BBI (cs-BBI). For anisotropic secondary domain growth, the ditopic modulators 4,4′-(2-phenyl-1*H*-imidazole-4,5-diyl)dibenzoic acid (M-ec_2_) or 4-(2-(4-(5-(4-carboxyphenyl)-4-phenyl-1*H*-imidazole-2-yl)phenyl)-5-phenyl-1*H*-imidazole-4-yl)benzoic acid (M-sc_2_) were added to the growth solutions to prepare either ec-BBI and or sc-BBI, respectively. PXRD confirmed that the resulting binary domain Zr-BBI MOFs were crystalline and isostructural to Zr-BBI (Fig. S24†). We note that the Zr-BBI-Br_2_ seeds were polydisperse in size. Therefore, for this system, comparing the crystal dimensions would not provide meaningful information regarding differences in domain growth between cs-, ec-, and sc-BBI products. We instead relied exclusively on SEM-EDS line-scans, conducted for Br and Zr, to reveal domain distribution (Fig. 4B). cs-BBI exhibited secondary domain growth in both the longitudinal and transverse directions, while ec-BBI and sc-BBI displayed growth exclusively along the transverse and longitudinal directions, respectively. Although the applicability of this method to two different MOFs does not imply generality, it does indicate that it could potentially be broadly applied to many non-cubic MOF systems.

### Anisotropic growth of ternary domain MOFs

An outstanding challenge in MOF chemistry involves the construction of arbitrarily complex multi-domain MOF systems.^10^ Having successfully created anisotropic binary domain MOFs, we were motivated to apply our strategy to achieve even greater degrees of MOF hierarchical complexity. Building from the established PCN-608-OMe system, we used cs-PCN, ec-PCN and sc-PCN as seeds for growth of Zr-based tertiary domains in either the absence or presence of the ditopic modulators. These syntheses resulted in 9 different ternary domain MOF products. Prior to preparing the ternary domain MOFs, we quantified the amount of M-ec_1_ within the ec-PCN MOF seeds. ^1^H NMR spectra of the dissolved seeds indicated a 0.07 : 1 modulator to ligand ratio, which we expected would negligibly affect tertiary domain growth.

**Fig. 5:**
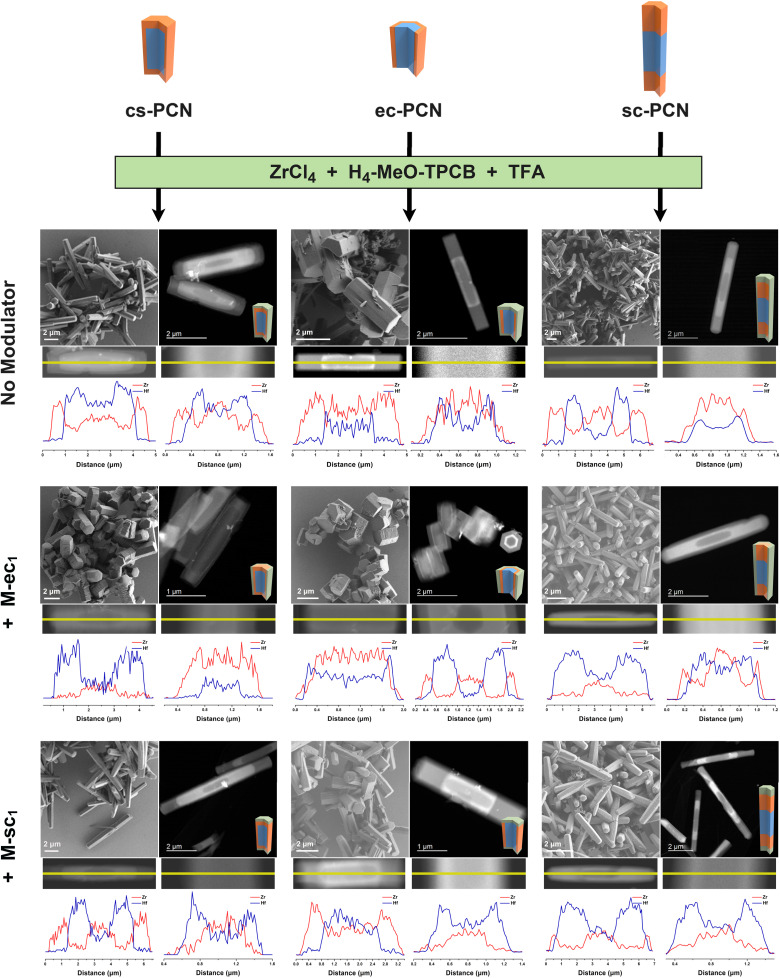
SEM, HAADF images and longitudinal (left) and transverse (right) STEM-EDS line-scans for nine ternary domain PCN MOFs synthesized with different modulators using binary cs-PCN, ec-PCN and sc-PCN as seeds.

HAADF imaging was again used to image the MOF domains (Fig. 5). In the absence of modulator, all three MOF products exhibit darker shell domains in the HAADF images, indicating the presence of Zr. Due to the larger size of the seed MOFs, shell growth in the longitudinal direction was more easily discernible. STEM-EDS line-scans, however (Fig. 5), clearly indicate both longitudinal and transverse tertiary domain growth, which further confirm that any small amount of ditopic modulator remaining in the binary MOF seeds does not affect tertiary domain growth. With the addition of M-ec_1_, transverse tertiary domain growth onto all three binary domain seeds was observed while minimal growth was observed in the longitudinal direction. Specifically, STEM-EDS line scans revealed distinct Zr signals at the outermost region along the transverse direction of the ternary domain MOFs, but Hf signal is strongest at the ends of the rod-like crystals. Similar conclusions can be drawn from HAADF images and EDS line-scans of the ternary domain MOFs formed with addition of M-sc_1_. In these cases, the resulting MOFs exhibited significant growth along the longitudinal directions but no observable growth in the transverse directions. In summary, we successfully synthesized nine different ternary domain MOFs with diverse anisotropic domain distributions by varying the type of ditopic modulator added to the synthesis.

## Conclusion

In this study, we demonstrated that modulators designed to selectively coordinate to specific crystalline facets can positionally direct the growth of secondary and tertiary MOF domains, resulting in the formation of AMD-MOFs. We systematically applied this strategy to two different MOF platforms, PCN-608-OMe and Zr-BBI, resulting in a diverse collection of hierarchically complex MOFs. In the case of PCN-608-OMe, we successfully applied multistep modulated syntheses to prepare various ternary domain MOFs *via* controlled anisotropic domain growth. These results underscore a robust and straightforward synthetic approach for designing and forming AMD-MOFs, which is critical for expanding access to such materials and ultimately more broadly exploring their properties and applications. Further development of these methods will investigate the effects of MOF seed morphology and modulator concentration in influencing secondary MOF domain growth and the formation of AMD-MOFs with other MOF platforms.

## Data availability

The data supporting this article have been uploaded as part of the ESI.†

## Author contributions

Y. H.: conceptualisation, investigation, methodology, formal analysis, writing original draft, writing review and editing; Z. L.: investigation, formal analysis; Z. M. S.: investigation, formal analysis; G. H.: investigation, formal analysis; N. L. R.: conceptualisation, funding acquisition, project administration, supervision, writing original draft, writing review and editing.

## Conflicts of interest

There are no conflicts to declare.

## Supplementary Material

SC-OLF-D4SC07985J-s001
