# Supplementary material for: Modulator approach for the design and synthesis of anisotropic multi-domain metal–organic frameworks
Source: Chem Sci. 2025 Mar 20;16(17):7442–9. doi: 10.1039/d4sc07985j (PMC11948342; doi:10.1039/d4sc07985j)
Supplement: SC-016-D4SC07985J-s001 [file SC-016-D4SC07985J-s001.pdf]

Supplementary Information (SI) for Chemical Science.
This journal is © The Royal Society of Chemistry 2025

Supporting Information for

Modulator Approach for the Design Synthesis of Anisotropic Multi-Domain Metal-Organic Frameworks

Yiwen He,[a] Zhehao Li,[a] Zoe M. Soilis,[a] Gefan He[a] and Nathaniel L. Rosi[ab]*

[a]Department of Chemistry, University of Pittsburgh, Pittsburgh, Pennsylvania, 15260.

[b]Department of Chemical and Petroleum Engineering, University of Pittsburgh, 3700 O'Hara Street, Pittsburgh, Pennsylvania 15261.

Table of Contents

1. General Methods 3
2. Syntheses and Characterizations of Organic Ligands 5
   4,4'-dimethoxybiphenyl-3,3',5,5'-tetra(phenyl-4-carboxylic acid) (H_4-MeO-TPCB) 5
   (1,1':3',1'':3'',1'''-quaterphenyl)-4,4'''-dicarboxylic acid (M-sc_1) 6
   (1,1',3',1''-terphenyl)-4,4''-dicarboxylic acid (M-ec_1) 7
   4,4',4'',4'''-(1,4-phenylenebis(1H-imidazole-2,4,5-triyl))tetrabenzoic acid (H_4-BBI) 8
   2,2'-(2,5-dibromo-1,4-phenylene)bis(1H-imidazole-4,5-dicarboxylic acid) (H_4-BBI-Br_2) 9
   4-(2-(4-(5-(4-carboxyphenyl)-4-phenyl-1H-imidazol-2-yl)phenyl)-5-phenyl-1H-imidazol-4-yl)benzoic acid (M-sc_2) 13
   4,4'-(2-phenyl-1H-imidazole-4,5-diyl)dibenzoic acid (M-ec_2) 17
3. Syntheses and Characterization of MOF Seed Crystallites 21
   PCN-608-OMe Seeds 21
   Metal Exchange Experiment with PCN-608-OMe Seeds 23
4. Syntheses and Characterization of Binary Domain MOFs 26
   Synthesis of cs-PCN 26
   Synthesis of ec-PCN 26
   Synthesis of sc-PCN 27
   Synthesis of cs-BBI 27
   Synthesis of ec-BBI 28
   Synthesis of sc-BBI 28
   ^{1}H NMR of ec-PCN 29
   PXRD Patterns of Binary Domain MOFs 30
   Dimensions of Binary Domain MOFs 31
5. Syntheses and Characterization of Ternary Domain MOFs 33
   Syntheses of Ternary Domain MOFs using cs-PCN Seeds 33
   Syntheses of Ternary Domain MOFs using ec-PCN Seeds 33
   Syntheses of Ternary Domain MOFs using sc-PCN Seeds 34
   PXRD Patterns of Ternary Domain MOFs 35
6. References 37

1. General Methods

All reagents and solvents were purchased through Fisher Scientific and used as received. See Table S1 for detailed information.

Table S1. Chemical information.

Name	CAS Number	Purity	Catalog #
N,N-Dimethylformamide	68-12-2	≥99.8 %	D1194
Ethanol	64-17-5	≥99.5%	7678004
Acetonitrile	75-05-8	≥99.9%	A9554
Trifluoroacetic acid	76-05-1	99%	AC139721000
Formic acid	64-18-6	≥99.0 %	A117
Zirconium (IV) chloride	10026-11-6	≥99.5%	221880
Hafnium (IV) chloride	13499-05-3	99.90%	590592
Tetrahydrofuran	109-99-9	≥99.9%	T3974
Potassium carbonate	584-08-7	≥99.0%	P208
Magnesium sulfate	10034-99-8	99%	01-337-186
Cesium carbonate	534-17-8	99%	AA1288718
Tetrakis(triphenylphosphine)palladium	14221-01-3	98%	A211391
1.4-dioxane	123-91-1	≥99.75%	AC326890010
Dichloromethane	75-09-2	>99.5%	D65100
Sodium hydroxide	1310-73-2	≥97.0%	S318500
3,3'-dibromo-1,1'-biphenyl	16400-51-4	>99.5%	NC2215880
(4-(methoxycarbonyl)phenyl)boronic acid	99768-12-4	98%	A397936
Palladium(II) acetate	3375-31-3	46.6-49.5% palladium (Pd) basis	683124
XPhos	564483-18-7	97%	A167774
Vitamin B1	59-43-8	98%	A117613
Dimethyl sulfoxide	67-68-5	99.90%	D128
Terephthalaldehyde	623-27-8	≥98.0%	T0010
Chloroform	67-66-3	>99.8%	AA32614K2
Ethyl acetate	141-78-6	≥99.5%	AA31344K7
Hexane	92112-69-1	≥98.5 %	H292
Sulfuric acid	7664-93-9	95.0 to 98.0 w/w %	A300
Acetic acid	64-19-7	100%	MAX00739
Acetic anhydride	108-24-7	≥97.0 %	A10-500
Chromium(VI) oxide	1333-82-0	>98+%	AA3646836
Ammonium acetate	631-61-8	≥97%	A637
Tetrahydrofuran	109-99-9	≤0.02% water	T397
HCl	7647-01-0	36.5-38.0%	AA33257P6
Benzaldehyde	100-52-7	≥99.0%	50-185-3894

Powder X-ray diffraction patterns were collected using a Bruker AXS D8 Discover powder diffractometer at 40 kV, 40 mA for Cu Kα (λ= 1.5406 Å) with a scan speed of 0.20 sec/step from 3.5 to 45° at a step size of 0.02°. The EVA program from the Bruker Powder Analysis Software package was used to perform background correction. Simulated powder patterns were calculated using Mercury 3.8 based on MOF crystal structures accepted by the Cambridge Crystallographic Data Centre.

^{1}H NMR and ^{13}C NMR spectra were obtained using Bruker Avance III 300/500 MHz spectrometers. Chemical shifts are presented in parts per million (ppm) using the residual solvent peak ($CDCl_3$ or DMSO-d_6) as references.

High resolution mass spectra (HRMS) were obtained on a Bruker Daltonics, Inc. APEXIII 7.0 TESLA FTMS instrument (ESI).

Fourier transform infrared (FTIR) spectra were recorded using the Thermo Fisher Nicolet Avatar FTIR spectrometer. The scan range was 4000–500 cm^{-1}. Absorptions are described as strong (s), medium (m), weak (w) and broad (br). Data was analyzed using the Omnic Software Package.

Scanning electron microscopy (SEM) data were collected using a ZEISS Sigma 500 VP scanning electron microscope. Samples were dispersed in 50:50 v/v ethanol/acetonitrile (EtOH/ACN) and drop cast on 12 mm carbon tape (Pelco image tabs, 260 μm carbon conductive tabs, prod#16084-20). The carbon tape was placed on SEM sample holder and dried under ambient conditions before SEM studies. ImageJ software was used for sizing.[1, 2]

High angle annular dark field (HAADF) imaging and scanning transmission electron microscopy - energy dispersive X-ray spectroscopy (STEM-EDS) studies were conducted on a JEOL JEM-2100F (PCN-608-0Me system) or ZEISS Sigma 500 VP SEM (Zr-BBI system). The STEM-EDS data was collected using 1024 channels from 0 to 20 keV. Zirconium line-scans were obtained using the Zr Kα1 line intensity at 15.7 keV. Hafnium line-scans were obtained using the Hf Lα1 line intensity at 7.9 keV. Bromine line-scans were obtained using the Br Kα1 line intensity at 11.9 keV. Samples were dispersed in 50:50 v/v EtOH/ACN and drop cast on TEM grids (Ted Pella Inc 200 mesh carbon film copper grids). The TEM grids were dried under ambient conditions before HAADF and STEM-EDS analyses.

2. Syntheses and Characterizations of Organic Ligands

4,4'-dimethoxybiphenyl-3,3',5,5'-tetra(phenyl-4-carboxylic acid) (H_4-MeO-TPCB)

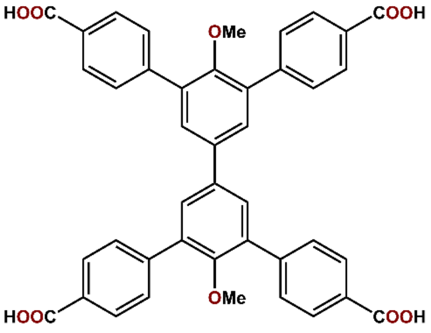

H_4-MeO-TPCB was synthesized following a literature protocol.[3]

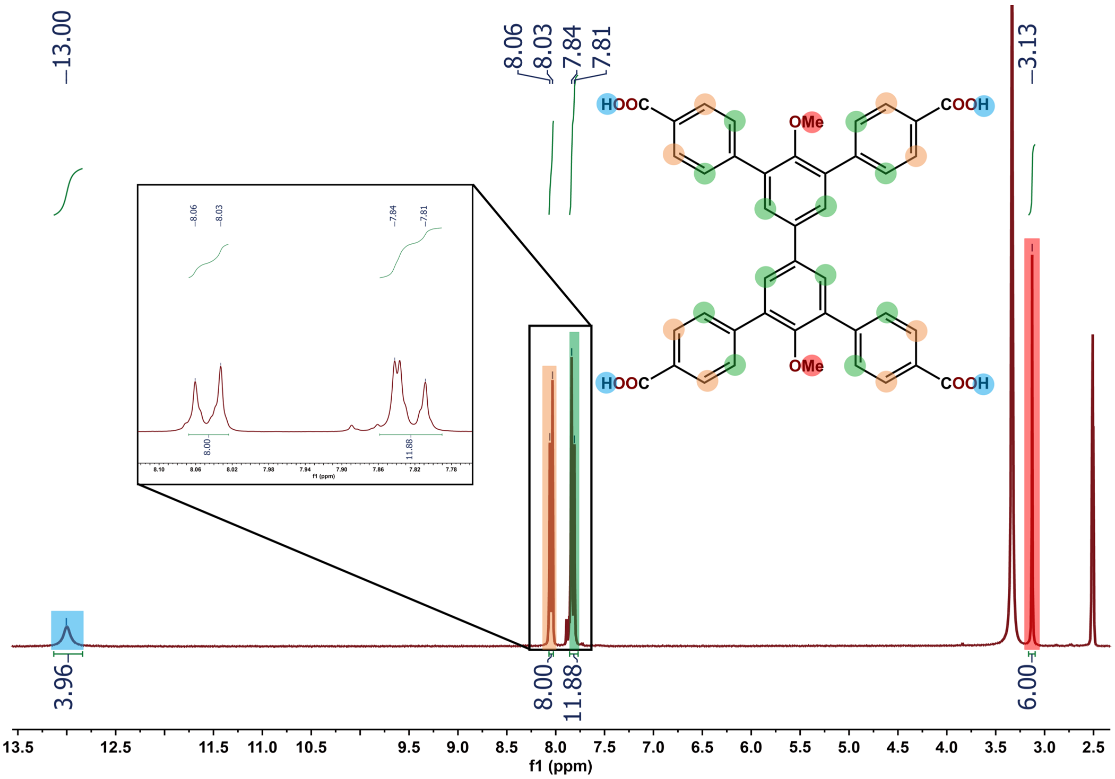

Figure S1. The ^{1}H NMR spectrum of H_4-MeO-TPCB.

(1,1':3',1'':3'',1'''-quaterphenyl)-4,4'''-dicarboxylic acid (M-sc₁)

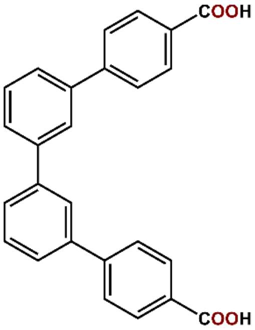

M-sc$_1$ was synthesized following a literature protocol.[4]

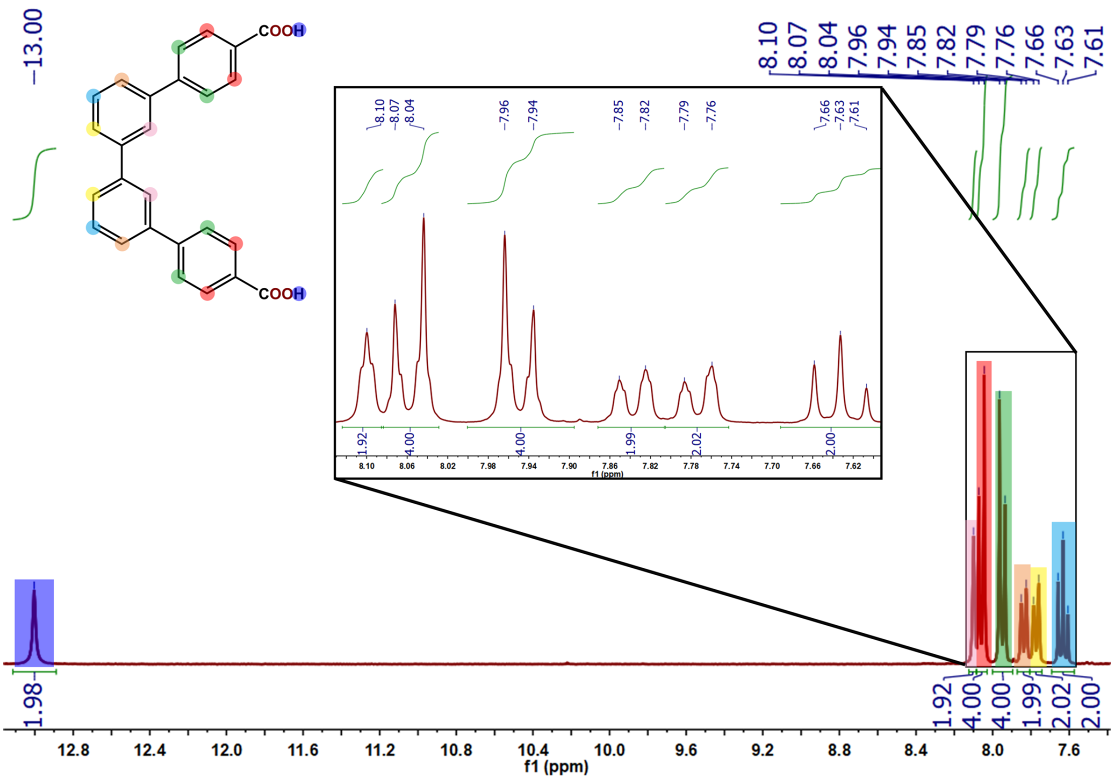

Figure S2. The ^{1}H NMR spectrum of **M-sc$_1$**.

(1,1',3',1''-terphenyl)-4,4''-dicarboxylic acid (M-ec₁)

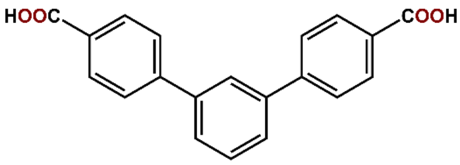

M-ec$_1$ was synthesized following a literature protocol.[5]

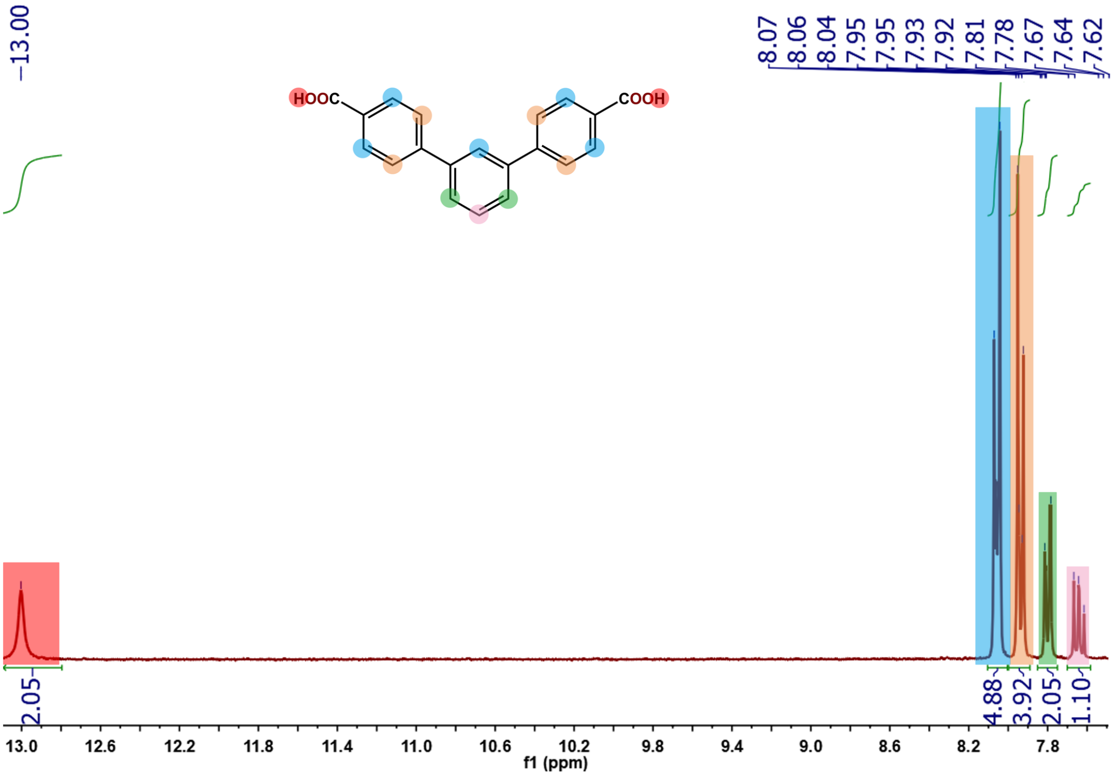

Figure S3. The ^{1}H NMR spectrum of **M-ec$_1$**.

4,4',4'',4'''-(1,4-phenylenebis(1H-imidazole-2,4,5-triyl))tetrabenzoic acid (H_4-BBI)

H_4-BBI was synthesized following a literature protocol.[6]

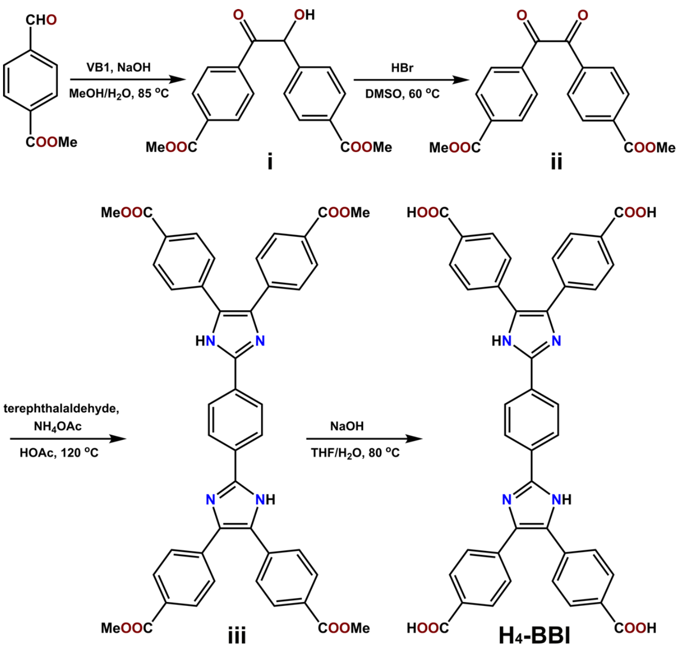

Scheme S1. Synthetic scheme for preparation of **H_4-BBI**.[6]

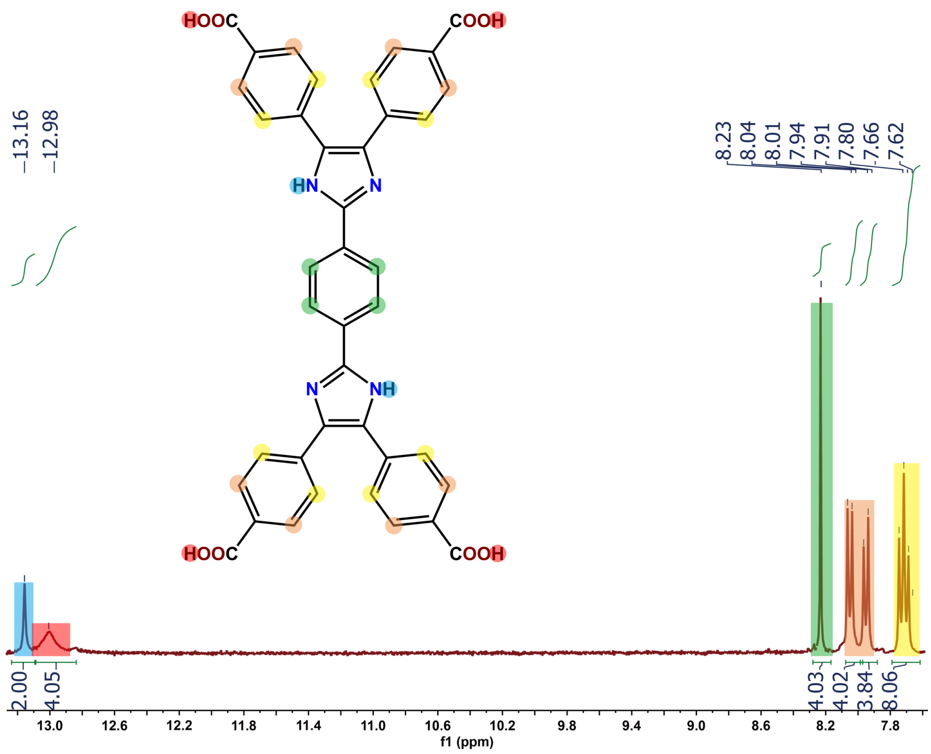

Figure S4. The ^{1}H NMR spectrum of **H_4-BBI**.

2,2'-(2,5-dibromo-1,4-phenylene)bis(1H-imidazole-4,5-dicarboxylic acid) (H_4-BBI-Br_2)

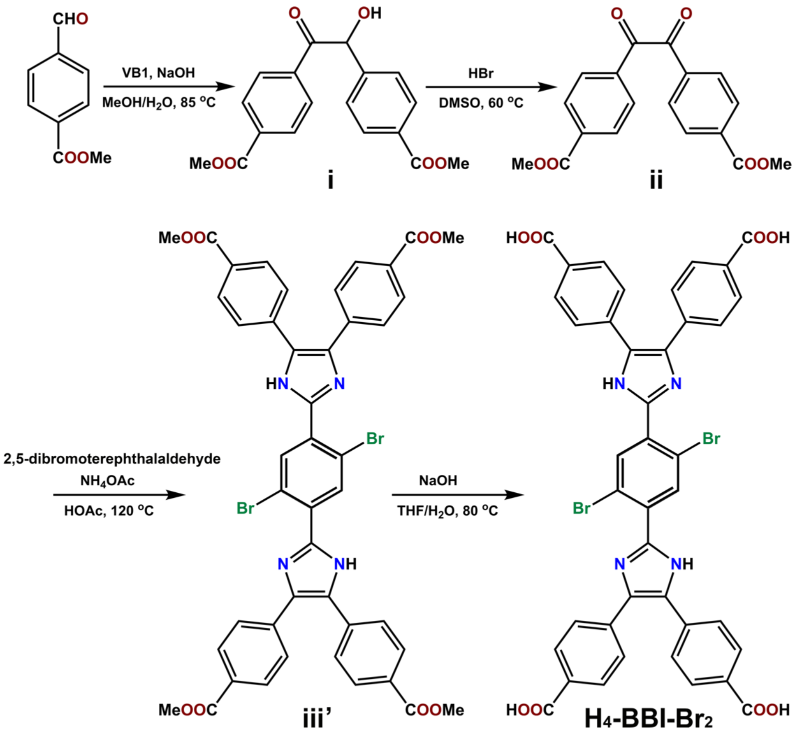

Scheme S2. Synthetic scheme for preparation of **H_4-BBI-Br_2**.

Synthesis of **i**:

Compound **i** was synthesized following a literature protocol.[6]

Synthesis of **ii**:

Compound **ii** was synthesized following a literature protocol.[6]

Synthesis of **iii'**:

The obtained product **ii** (3.3 g, 10 mmol), 2,5-dibromoterephthalaldehyde (0.99 g, 3.4 mmol), and ammonium acetate (5.2 g, 67 mmol) was dissolved in acetic acid (45 mL). The solution was heated and refluxed under N_2 atmosphere for 24 h. After completion of the reaction, the mixture was poured into ice cold water. The precipitate was collected by filtration and washed with water. The crude product was washed with $CHCl_3$/MeOH (50:50) mixture to afford **iii'** as a yellow solid in 80% yield.

Synthesis of **H_4-BBI-Br_2**:

The obtained tetraester **iii'** (1.84 g, 2.68 mmol) and NaOH (3.20 g, 79.6 mmol) was placed in a mixture of THF (25 mL) and H_2O (70 mL). The mixture was heated and refluxed for 24 h. After cooling to room temperature, THF was removed via vacuum. The remaining aqueous solution was acidified with 2 M HCl to give a yellow precipitate. The precipitate was filtered, washed with water, and dried. The obtained product was further purified by washing with ethyl acetate and hexane to afford **H_4BBI-Br_2** as a yellow solid in 95% yield. ^{1}H NMR (500 MHz, DMSO-d_6): δ =13.16 (s, 2 N–H), 12.97 (s, 4H), 8.23 (s, 2H), 8.025 (d, J = 8.4 Hz, 4H), 7.925 (d, J = 8.4 Hz, 4H), 7.69 (m, J = 8.8 Hz, 8H). ^{13}C NMR (126 MHz, DMSO-d_6): δ = 167.6, 167.4, 144.2, 139.4, 137.8, 136.2, 135.0, 133.1, 130.6, 130.3, 130.0, 129.5, 129.4, 128.8, 127.7, 120.5. HRMS (ESI) calculated for $C_{40}H_{23}O_8N_4Br_2$ $[M-H]^-$: 844.9877, found: 844.9891.

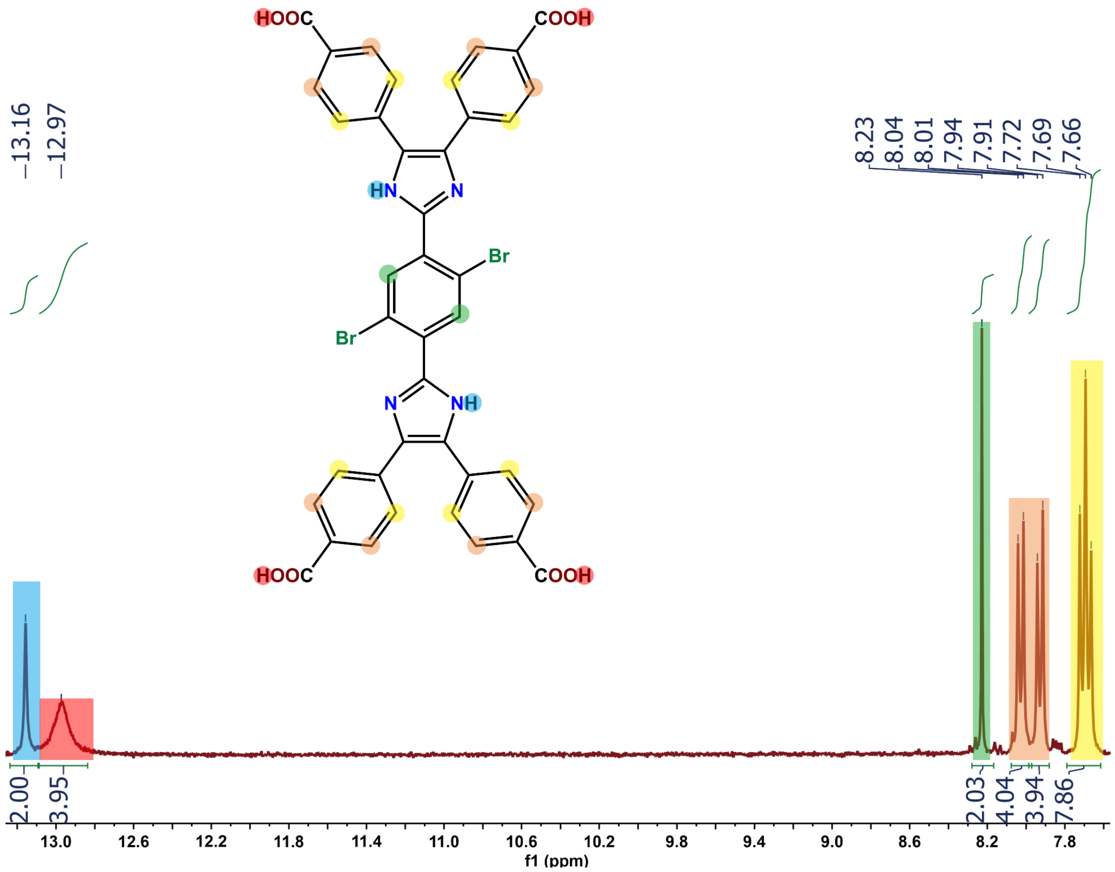

Figure S5. The ^{1}H NMR spectrum of **H_4-BBI-Br_2**.

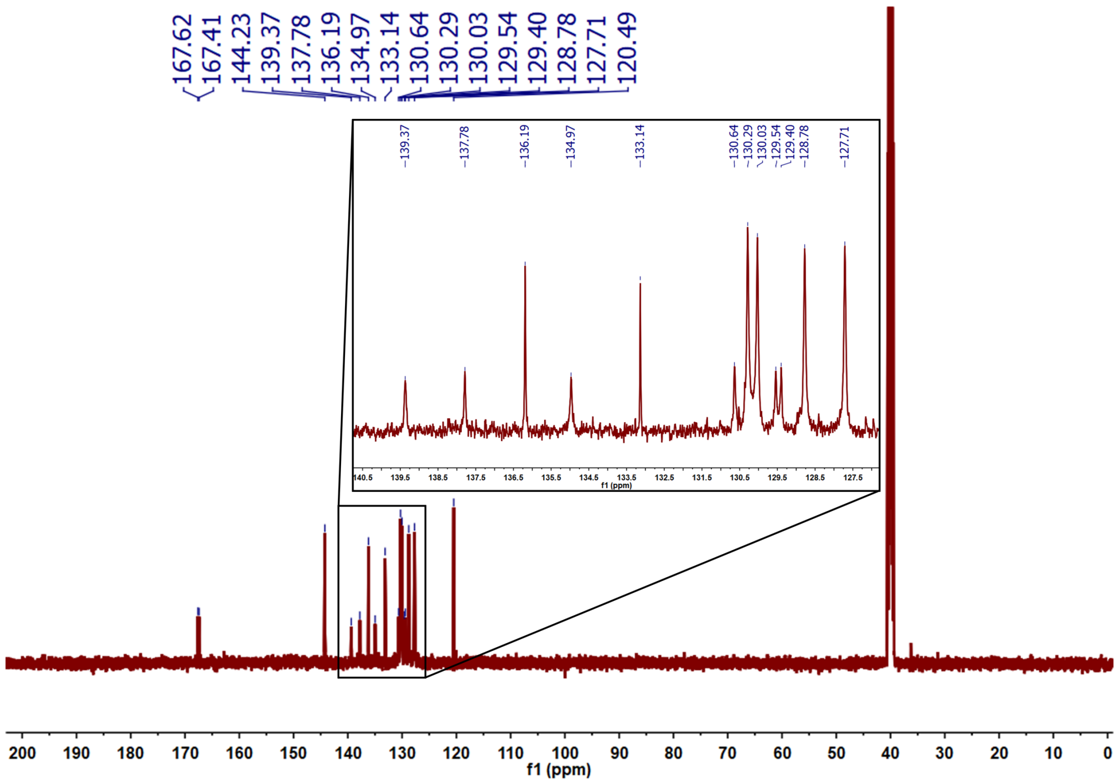

Figure S6. The ^{13}C NMR spectrum of **H_4-BBI-Br_2**.

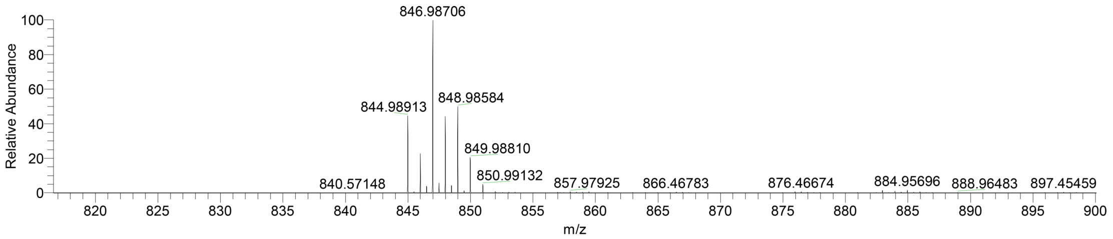

T: FTMS - p ESI Full ms [100.0000-1000.0000]

m/z= 844.40999-845.45701

m/z	Intensity	Relative	Theo. Mass	Delta (ppm)	Composition
844.98913	28318852.0	100.00	844.98772	1.68	$C_{40}H_{23}O_8N_4Br_2$

Figure S7. The HRMS spectrum of **H_4-BBI-Br_2**.

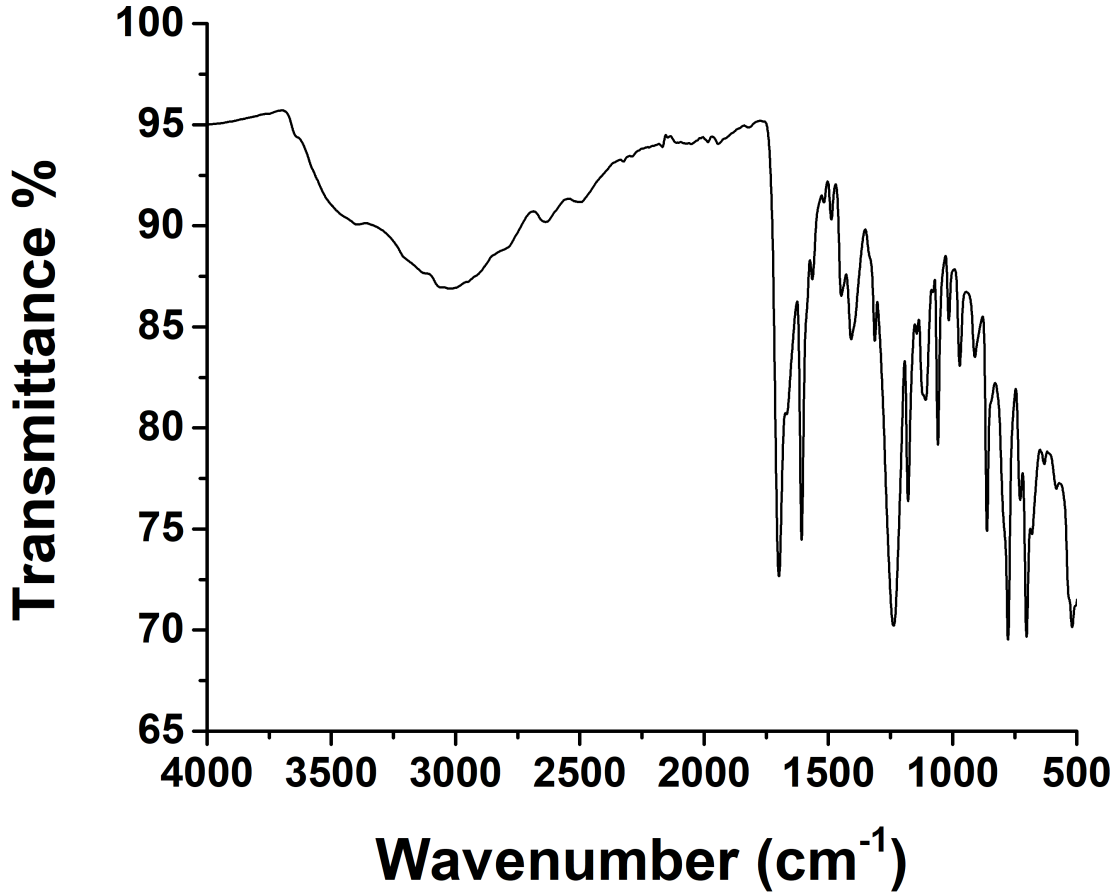

Figure S8. The FTIR spectrum of **H_4-BBI-Br_2** (4000-500 cm^{-1}): 3017 (br), 1699 (s), 1607 (s), 1408 (w), 1238 (s), 1178 (m), 1108 (w), 1058 (m), 861 (m), 777 (s), 702 (s), 519 (s).

4-(2-(4-(5-(4-carboxyphenyl)-4-phenyl-1H-imidazol-2-yl)phenyl)-5-phenyl-1H-imidazol-4-yl)benzoic acid (M-sc$_2$)

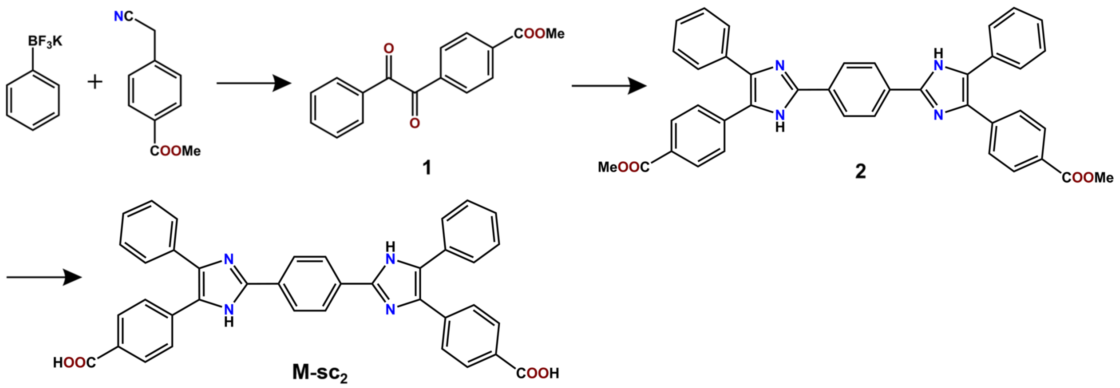

Scheme S3. Synthetic scheme for preparation of **M-sc$_2$**.

Synthesis of **1**:

Compound **1** was synthesized following a literature protocol.[7]

Synthesis of **2**:

The obtained product **1** (2.7 g, 10 mmol), terephthalaldehyde (0.45 g, 3.4 mmol), and ammonium acetate (5.2 g, 67 mmol) were dissolved in acetic acid (45 mL). The solution was heated and refluxed under N_2 atmosphere for 24 h. After completion of the reaction, the mixture was poured into ice water. The precipitate was collected by filtration and washed with water. The crude product was washed with a $CHCl_3$/methanol (50:50 v/v $CHCl_3$/MeOH) mixture to afford **2** as a light yellow solid in 65% yield. HRMS (ESI) calculated for $C_{40}H_{31}O_4N_4$ $[M+H]^+$: 631.2340, found: 631.2344.

Synthesis of **M-sc₂**:

The obtained compound **2** (2.7 g, 4.2 mmol) and NaOH (1.6 g, 40 mmol) was placed in a mixture of THF (15 mL) and H_2O (45 mL). The mixture was heated and refluxed for 24 h. After cooling to room temperature, THF was removed via vacuum. The remaining aqueous solution was acidified with 2 M HCl to give a light yellow precipitate. The precipitate was filtered, washed with water, and dried. The obtained product was further purified by washing with ethyl acetate and hexane to afford **M-sc$_2$** as a light yellow solid in 92% yield. ^{1}H NMR (500 MHz, DMSO-d_6): δ =12.96 (s, 4H), 8.28 (s, 4H), 7.97 (s, 4H), 7.74 (d, J = 7.5 Hz, 4H), 7.62 (d, J = 7.5 Hz, 4H), 7.51 (s, 6H). ^{13}C NMR (126 MHz, DMSO-d_6): δ =167.6, 146.1, 130.3, 130.1-125.3, 126.0. HRMS (ESI) calculated for $C_{38}H_{25}O_4N_4$ [M-H]$^-$: 601.1870, found: 601.1864.

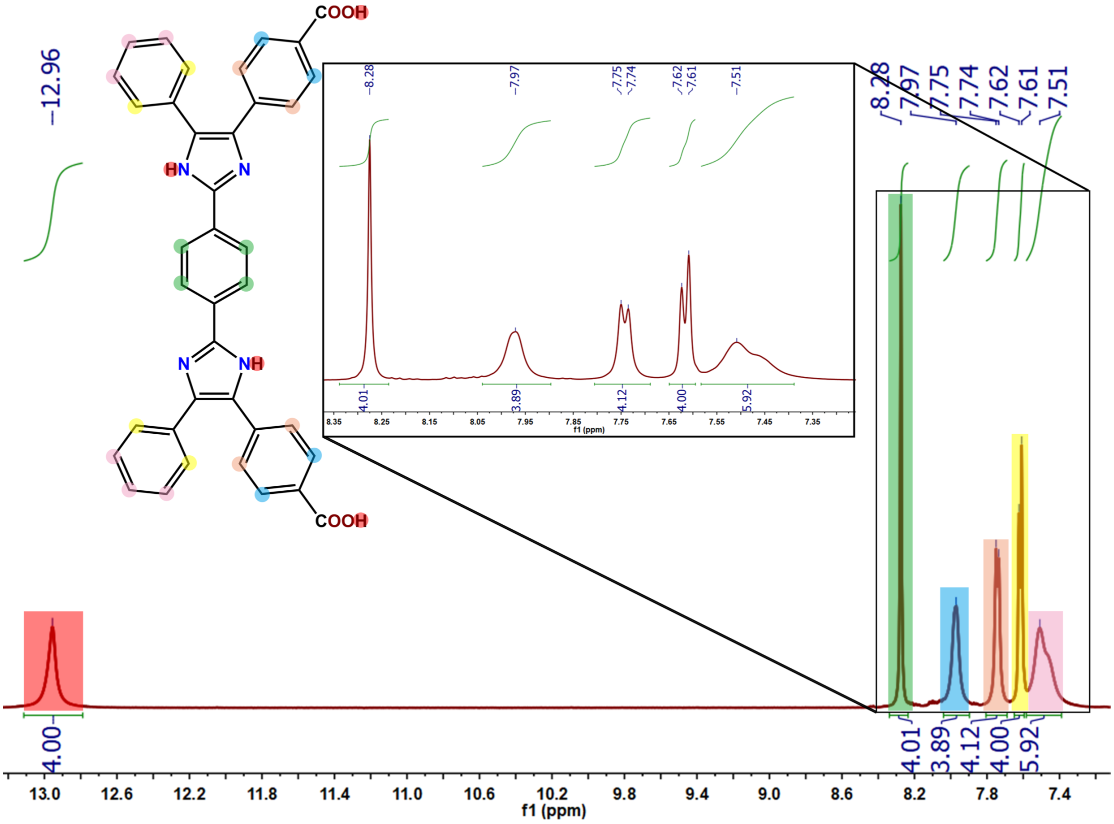

Figure S9. The ^{1}H NMR spectrum of **M-sc$_2$**.

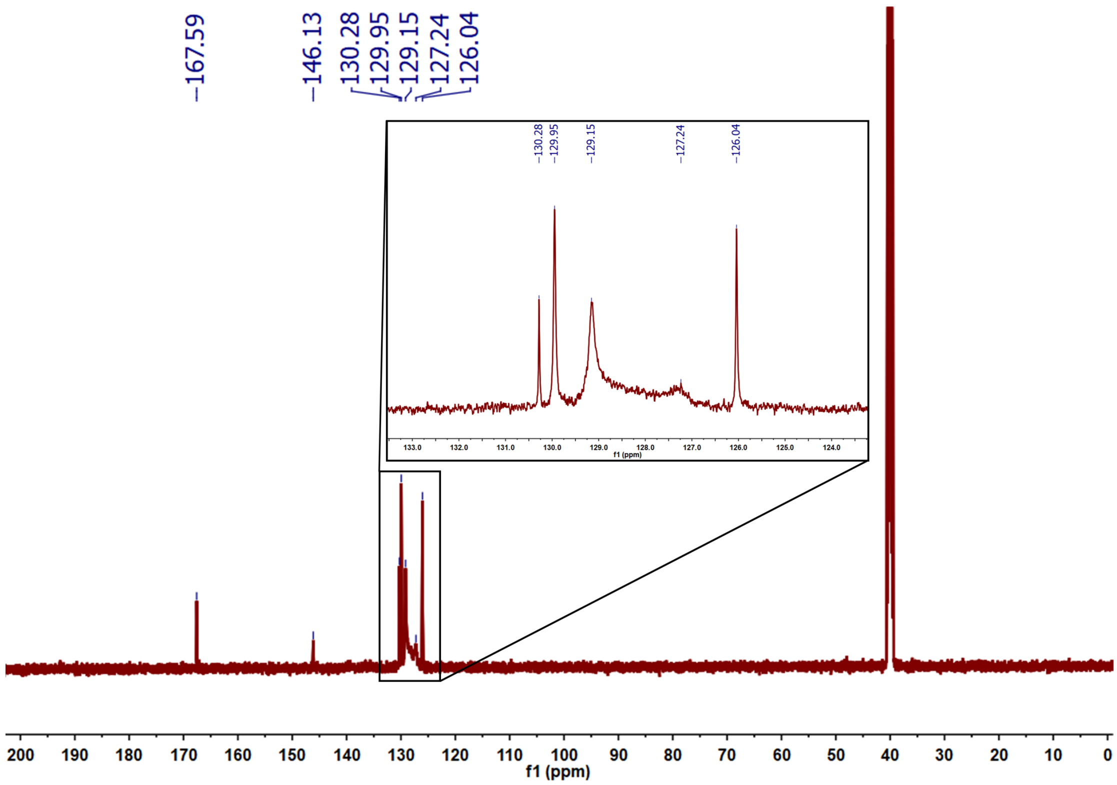

Figure S10. The ^{13}C NMR spectrum of **M-sc$_2$**.

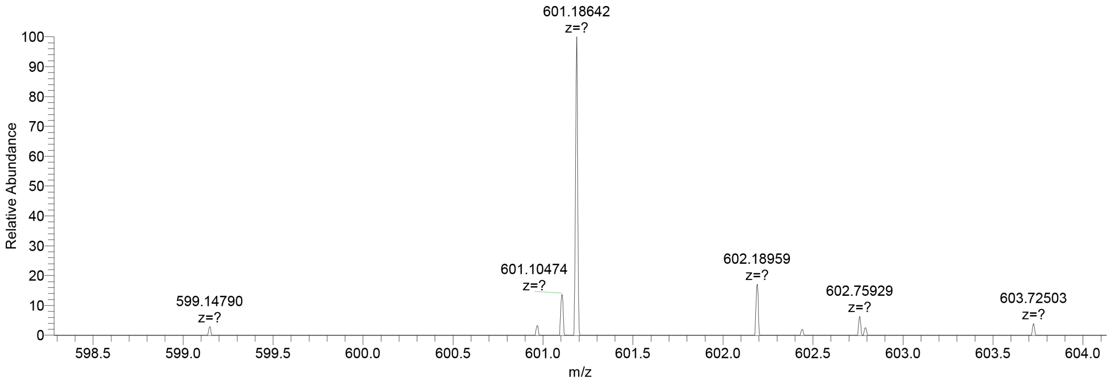

m/z= 598.53201-604.20368

m/z	Intensity	Relative	Theo. Mass	Delta (ppm)	Composition
601.18642	92644.9	100.00	601.18703	-1.02	$C_{38}H_{25}O_4N_4$

Figure S11. The HRMS spectrum of **M-sc$_2$**.

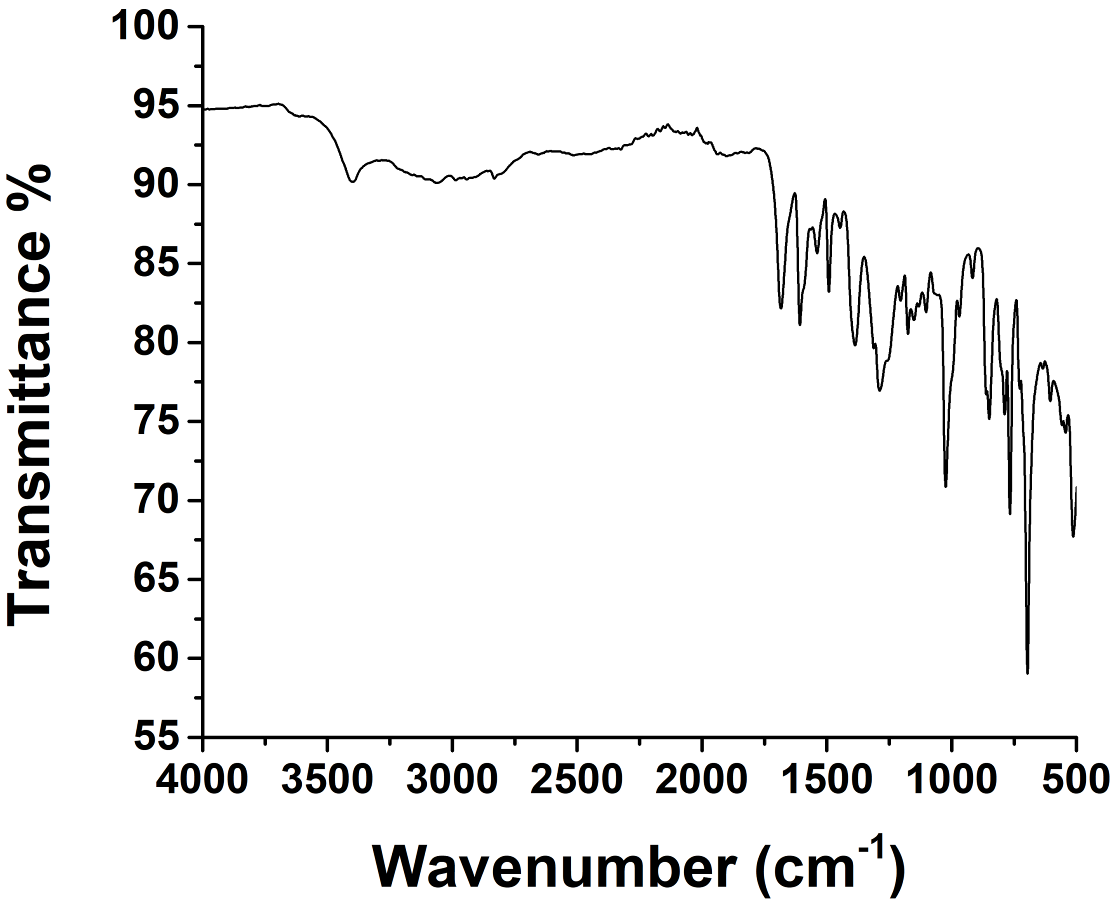

Figure S12. The FTIR spectrum of **M-sc$_2$** (4000-500 cm^{-1}): 3398 (w), 3062 (br), 1684 (m), 1607 (m), 1539 (w), 1491 (m), 1387 (m), 1289 (m), 1103 (w), 1023 (s), 848 (m), 767 (s), 696 (s), 605 (w), 545 (w), 512 (s).

4,4'-(2-phenyl-1H-imidazole-4,5-diyl)dibenzoic acid (M-ec₂)

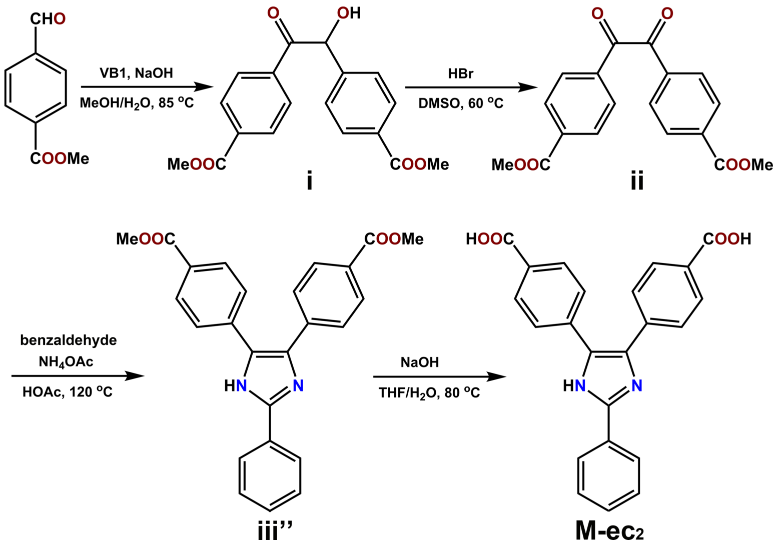

Scheme S4. Synthetic scheme for preparation of **M-ec$_2$**.

Synthesis of **i**:

Compound **i** was synthesized following a literature protocol.[6]

Synthesis of **ii**:

Compound **ii** was synthesized following a literature protocol.[6]

Synthesis of **iii''**:

The obtained product **ii** (3.3 g, 10 mmol), benzaldehyde (0.82g, 7.7 mmol), and ammonium acetate (5.2 g, 67 mmol) was dissolved in acetic acid (45 mL). The solution was heated and refluxed under N_2 atmosphere for 24 h. After completion of the reaction, the mixture was poured into ice cold water. The precipitate was collected by filtration and washed with water. The crude product was washed with $CHCl_3$/MeOH (50:50) mixture to afford **iii''** as off-white solid in 95% yield.

Synthesis of **M-ec₂**:

The obtained ester **iii''** (2.21 g, 5.36 mmol) and NaOH (3.20 g, 79.6 mmol) was placed in the mixture of THF (25 mL) and H_2O (70 mL). The mixture was heated and refluxed for 24 h. After cooling to room temperature, THF was removed in vacuum. The remaining aqueous solution was acidified with 2 M HCl to give off-white precipitate. The precipitate was filtered, washed with water and dried. The obtained product was further purified by washing with ethyl acetate and hexane to afford **M-ec$_2$** as off-white solid in 90% yield. ^{1}H NMR (500 MHz, DMSO-d_6): δ =13.01 (s, 3H), 8.17 (d, J = 7.5 Hz, 2H), 8.01 (s, 4H), 7.72 (d, J = 8.2 Hz, 4H), 7.56 (t, J = 7.6 Hz, 2H), 7.46 (t, J =7.4 Hz, 1H). ^{13}C NMR (126 MHz, DMSO-d_6): δ =167.5, 147.2, 130.4, 130.1,129.2, 128.8-127.3, 125.9. HRMS (ESI) calculated for $C_{23}H_{15}O_4N_2$ [M-H]$^-$: 383.1026, found: 383.1034.

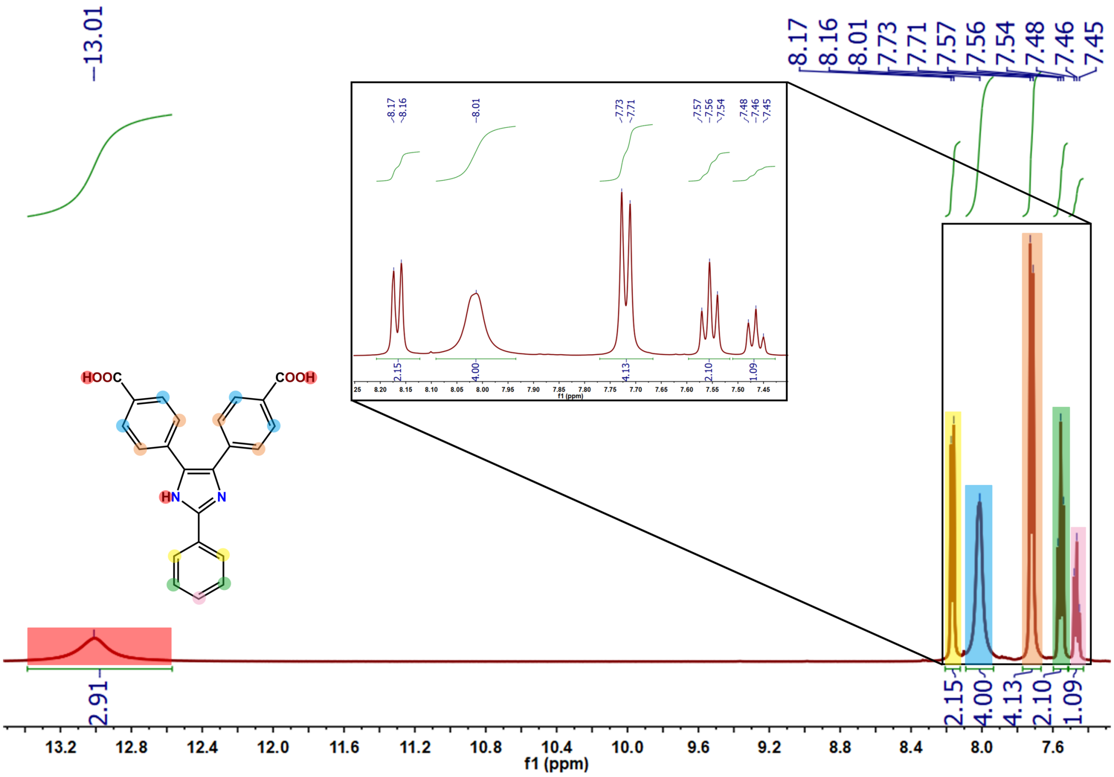

Figure S13. The ^{1}H NMR spectrum of **M-ec$_2$**.

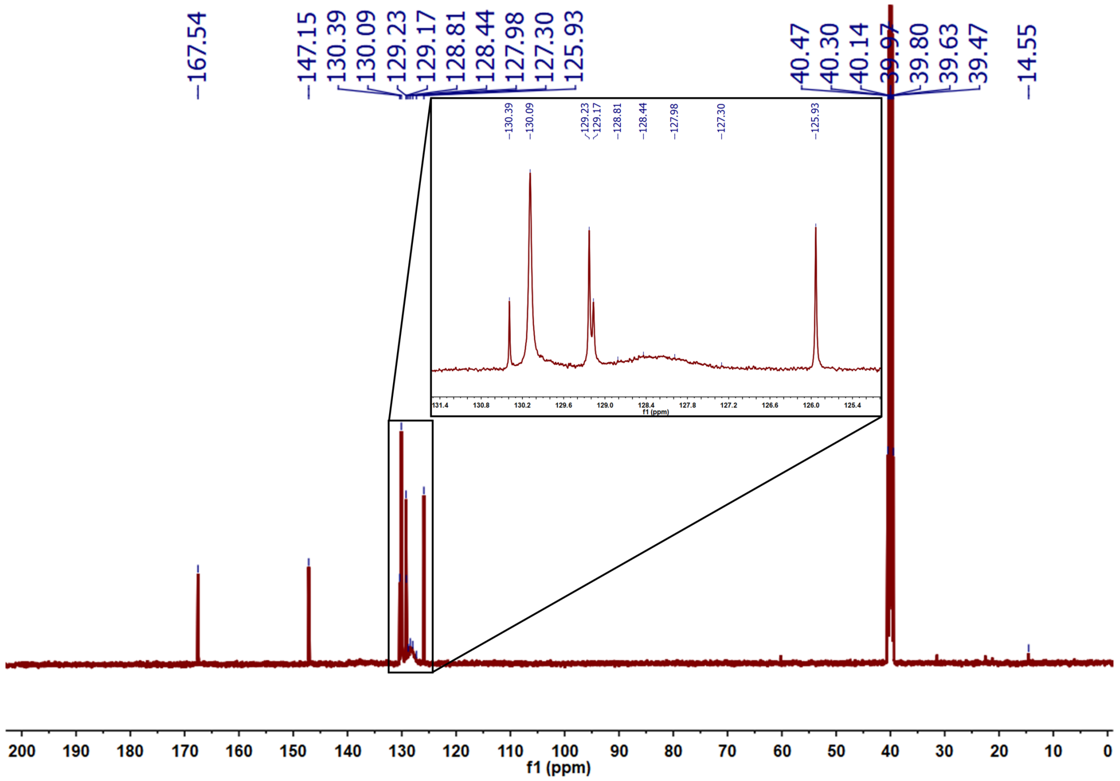

Figure S14. The ^{13}C NMR spectrum of **M-ec$_2$**.

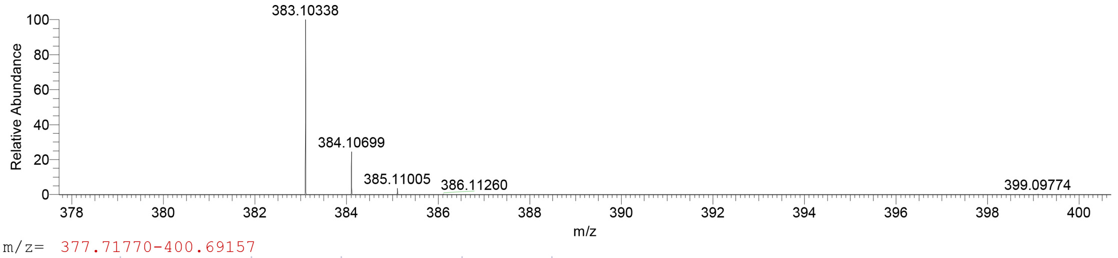

m/z= 377.71770-400.69157

m/z	Intensity	Relative	Theo. Mass	Delta (ppm)	Composition
383.10338	195043856.0	100.00	383.10263	1.96	$C_{23}H_{15}O_4N_2$

Figure S15. The HRMS spectrum of **M-ec$_2$**.

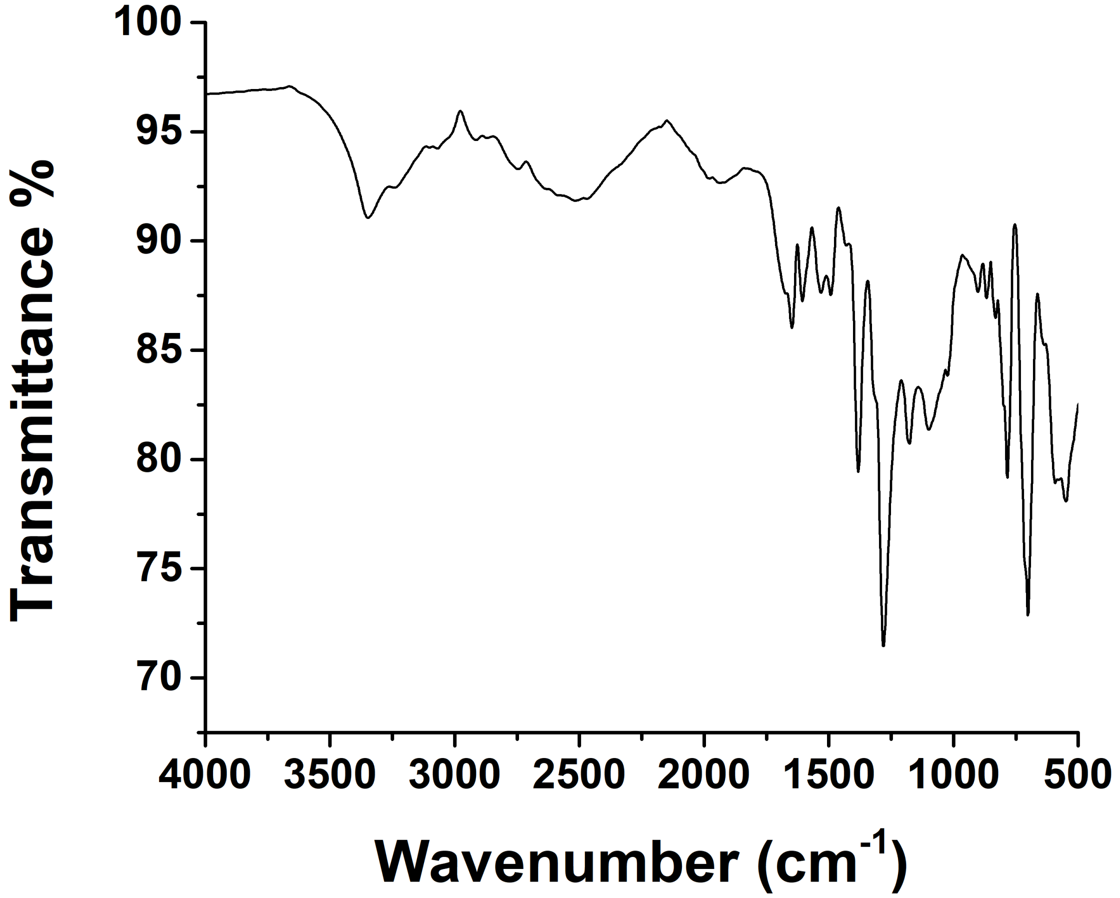

Figure S16. The FTIR spectrum of **M-ec$_2$** (4000-500 cm^{-1}): 3347 (br), 2517 (br), 1650 (w), 1603 (w), 1534 (w), 1493 (w), 1381 (m), 1280 (s), 1179 (m), 1097 (m), 903 (w), 787 (m), 699 (s), 552 (m).

3. Syntheses and Characterization of MOF Seed Crystallites

PCN-608-OMe Seeds

To a 20 mL Pyrex vial, $ZrCl_4$ (30 mg, 0.13 mmol), H_4-MeO-TPCB (15 mg, 0.022 mmol), formic acid (1.5 mL), and 15 mL dimethylformamide (DMF) were added. After sonication for 5 min to dissolve $ZrCl_4$ and H_4-MeO-TPCB, the vial was capped and placed in a 120 ºC oven for 20 h. After cooling to room temperature, the reaction suspension was then centrifuged at 6,000 rpm for 3 min to obtain ~30 mg of white solid. The solid was washed with fresh DMF (16 mL each time, 4x) and dispersed in DMF (1 mL). The prepared PCN-608-OMe/DMF suspension was used for characterization and subsequent experimental procedures.

0.05 mL PCN-608-OMe/DMF suspension was washed with MeOH (4 mL each time, 3x) and dispersed in 50:50 v/v ACN/EtOH (total volume 1 mL) for SEM analysis.

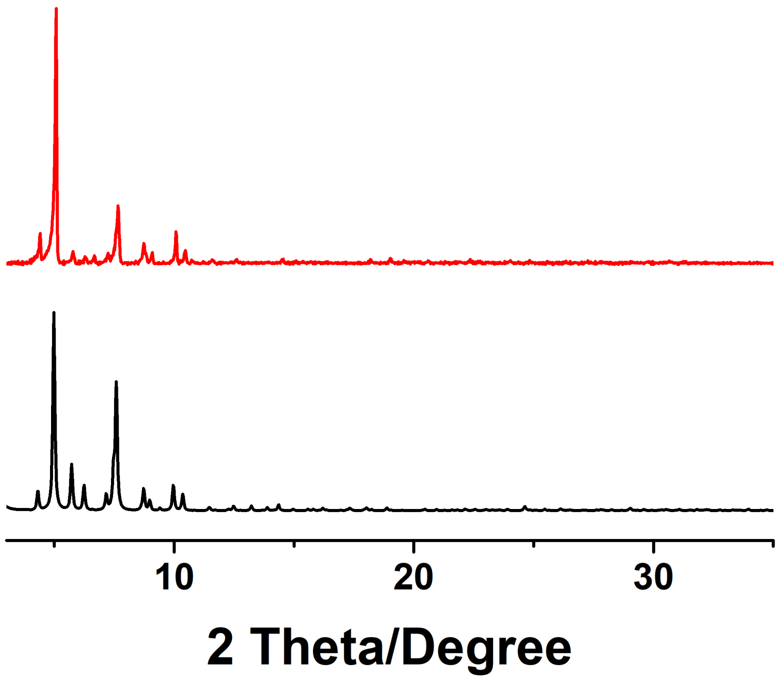

Figure S17. Simulated PXRD pattern of PCN-608 (black) and experimental PXRD pattern of as-synthesized PCN-608-OMe seed crystallites (red).

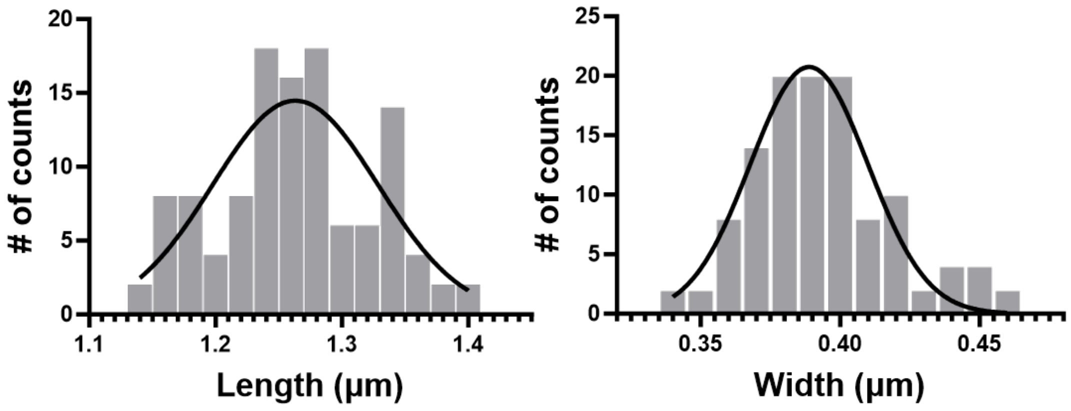

Figure S18. Length and width distributions of PCN-608-OMe seed crystallites fitted with Gaussian distribution curves (n=100 counts). The fit was performed using nonlinear regression.

Table S2. Average length, width and aspect ratio of PCN-608-OMe seed crystallites (n=100 counts).

**Length**	1.26±0.05 μm
**Width**	0.39±0.02 μm
**Aspect Ratio**	3.22±0.13

Metal Exchange Experiment with PCN-608-OMe Seeds

To a 20 mL Pyrex vial, $HfCl_4$ (2 mg, 0.006 mmol), trifluoroacetic acid (TFA, 0.16 mL), 10 mL DMF and PCN-608-OMe/DMF suspension (0.1 mL, as prepared in previous section) were added. After sonication for 5 min to dissolve $HfCl_4$, the vial was capped and placed in a 120 ºC oven for 20h. After cooling to room temperature, the reaction suspension was then centrifuged at 6,000 rpm for 3 min to obtain a white solid. The solid was washed with fresh DMF (10 mL each time, 4x) and dispersed in DMF (1 mL). 0.10 mL MOF/DMF suspension was washed with MeOH (4 mL each time, 3x) and dispersed in 50:50 v/v ACN/EtOH (total volume 1 mL) for STEM-EDS analysis.

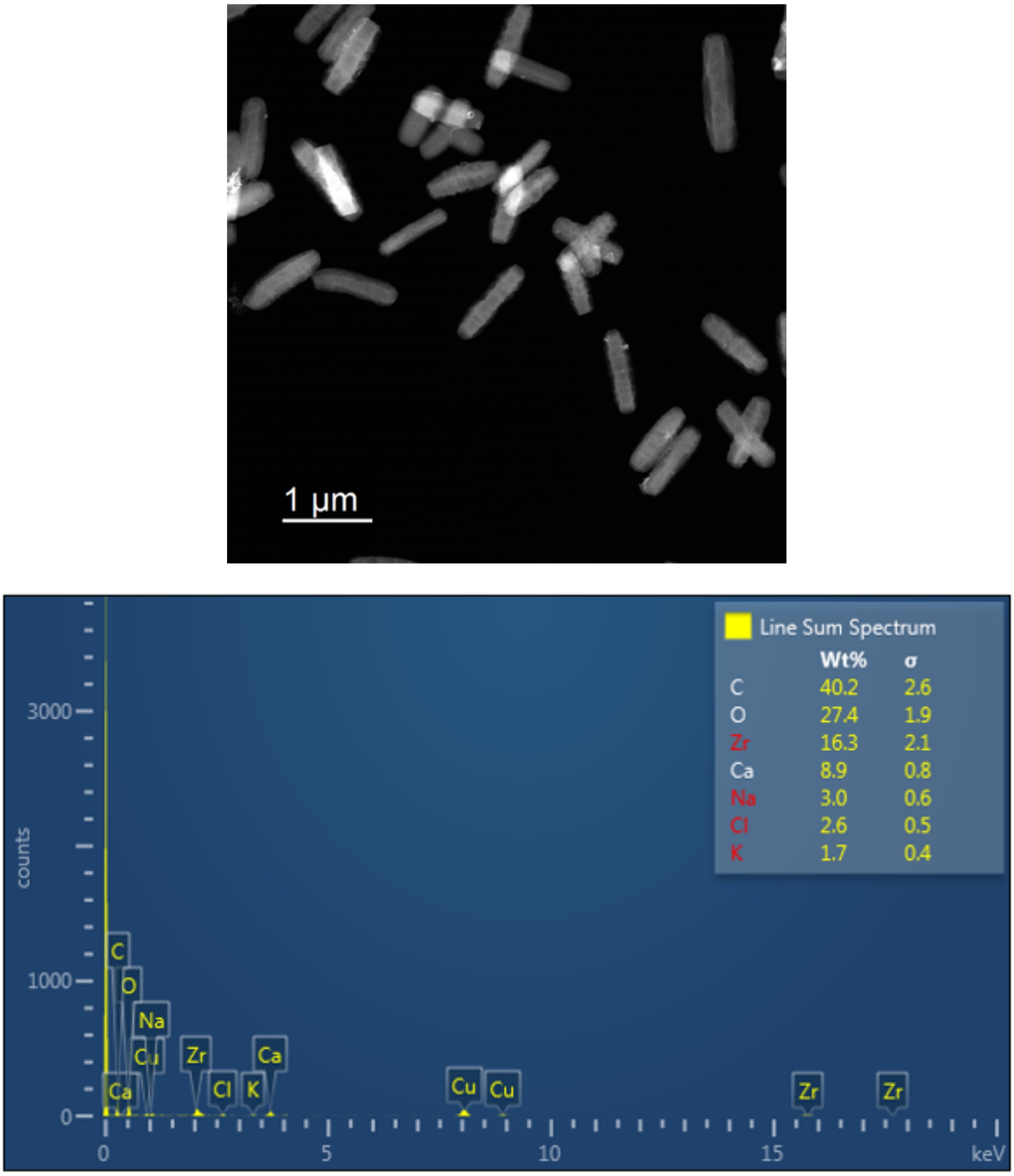

Figure S19. HAADF image of PCN-608-OMe(top) and EDS sum spectrum (bottom) of the surveyed area after metal exchange experiment.

Zr-BBI-Br_2 Seeds

To a 20 mL Pyrex vial, $ZrCl_4$ (32 mg, 0.14 mmol), H_4-BBI-Br_2 (20 mg, 0.024 mmol), formic acid (2.0 mL), and 10 mL DMF were added. After sonication for 5 min to dissolve $ZrCl_4$ and H_4-BBI-Br_2, the vial was capped and placed in a 100 °C oven for 20 h. After cooling to room temperature, the reaction suspension was then centrifuged at 6,000 rpm for 3 min to obtain ~35 mg of yellow solid. The solid was washed with fresh DMF (16 mL each time, 4x) and dispersed in DMF (1 mL). The prepared Zr-BBI-Br_2/DMF suspension was used for characterization and subsequent experimental procedures.

0.05 mL Zr-BBI-Br_2/DMF suspension was washed with MeOH (4 mL each time, 3x) and dispersed in 50:50 v/v ACN/EtOH (total volume 1 mL) for SEM analysis.

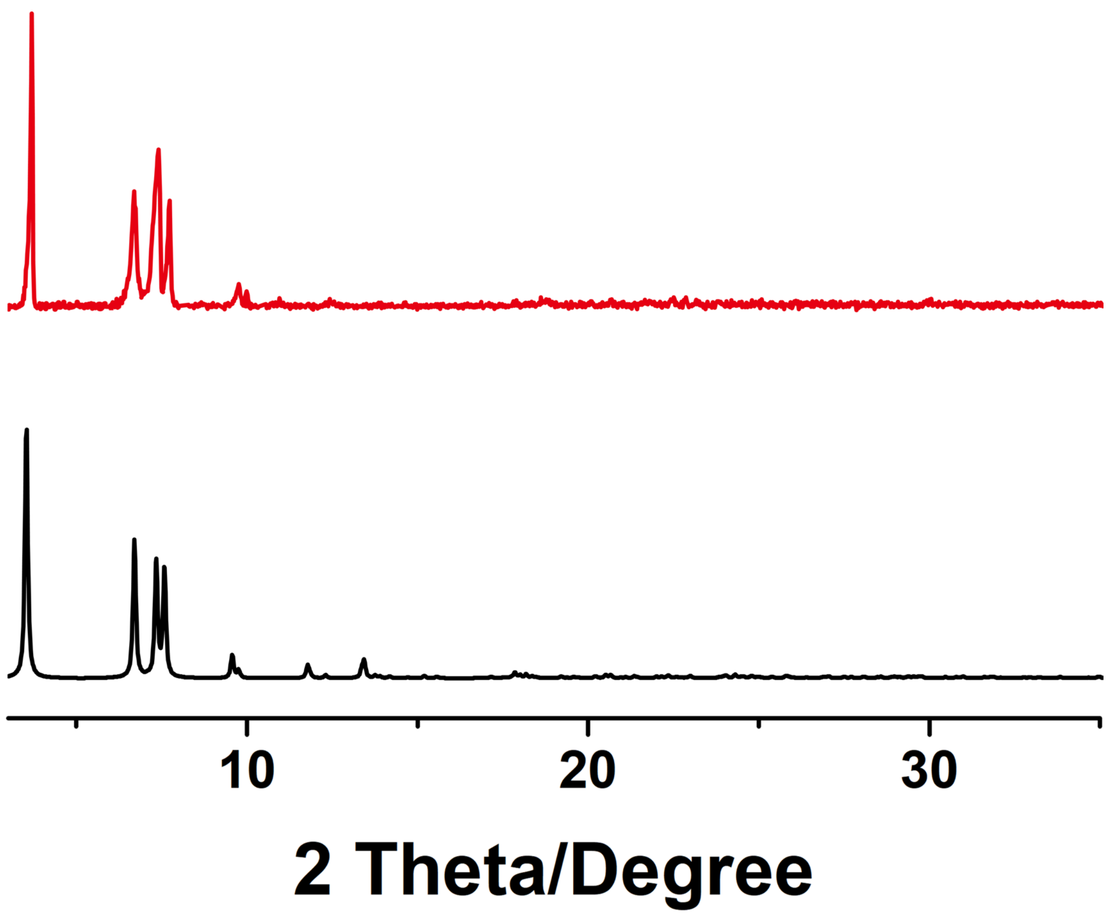

Figure S20. Simulated PXRD pattern of Zr-BBI (black) and experimental PXRD pattern of as-synthesized Zr-BBI-Br_2 seed crystallites (red).

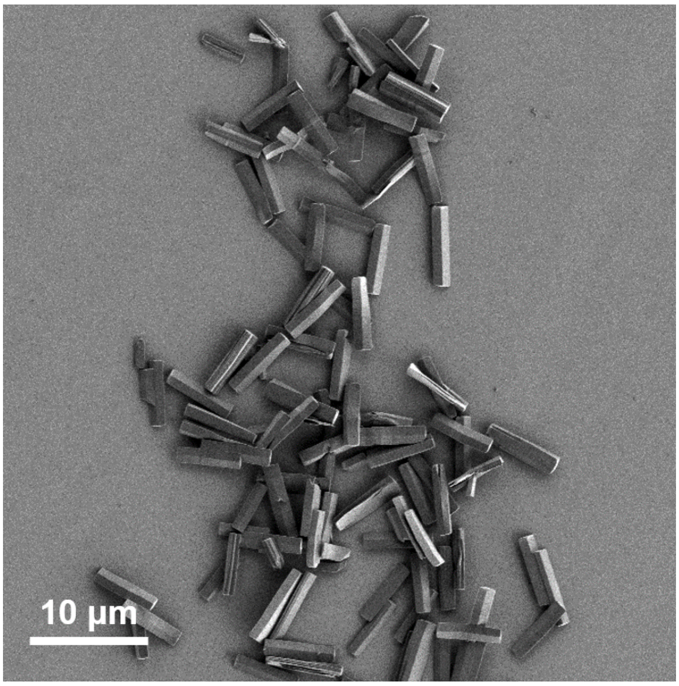

Figure S21. SEM image of Zr-BBI-Br_2 seed crystallites.

4. Syntheses and Characterization of Binary Domain MOFs

Synthesis of cs-PCN

To a 20 mL Pyrex vial, $HfCl_4$ (2 mg, 0.006 mmol), H_4-MeO-TPCB (2 mg, 0.003 mmol), trifluoroacetic acid (TFA, 0.16 mL), 10 mL DMF, and PCN-608-OMe/DMF suspension (0.1 mL, as prepared in previous section) were added. After sonication for 5 min to disperse seed crystals and dissolve $HfCl_4$ and H_4-MeO-TPCB, the vial was capped and placed in a 120 ºC oven for 20 h. After cooling to room temperature, the reaction suspension was then centrifuged at 6,000 rpm for 3 min to obtain ~4.5 mg of white solid. The solid was washed with fresh DMF (10 mL each time, 4x) and dispersed in DMF (1 mL). The prepared cs-PCN/DMF suspension was used for characterization and subsequent experimental procedures.

0.10 mL cs-PCN/DMF suspension was washed with MeOH (4 mL each time, 3x) and dispersed in 50:50 v/v ACN/EtOH (total volume 1 mL) for SEM, HAADF, and STEM-EDS analysis.

Synthesis of ec-PCN

To a 20 mL Pyrex vial, $HfCl_4$ (2 mg, 0.006 mmol), H_4-MeO-TPCB (2 mg, 0.003 mmol), trifluoroacetic acid (TFA, 0.20 mL), M-ec_1 (12 mg, 0.038 mmol), 10 mL DMF, and PCN-608-OMe/DMF suspension (0.1 mL, as prepared in previous section) were added. After sonication for 5 min to disperse seed crystals and dissolve $HfCl_4$, H_4-MeO-TPCB, and M-ec_1, the vial was capped and placed in a 120 ºC oven for 20 h. After cooling to room temperature, the reaction suspension was then centrifuged at 6,000 rpm for 3 min to obtain ~4.5 mg of white solid. The solid was washed with fresh DMF (10 mL each time, 4x) and dispersed in DMF (1 mL). The prepared ec-PCN/DMF suspension was used for characterization and subsequent experimental procedures.

0.10 mL ec-PCN suspension was washed with MeOH (4 mL each time, 3x) and dispersed in 50:50 v/v ACN/EtOH (total volume 1 mL) for SEM, HAADF, and STEM-EDS analysis.

Synthesis of sc-PCN

To a 20 mL Pyrex vial, $HfCl_4$ (2 mg, 0.006 mmol), H_4-MeO-TPCB (2 mg, 0.003 mmol), trifluoroacetic acid (TFA, 0.20 mL), M-sc_1 (15 mg, 0.038 mmol), 10 mL DMF, and PCN-608-OMe/DMF suspension (0.1 mL, as prepared in previous section) were added. After sonication for 5 min to disperse seed crystals and dissolve $HfCl_4$, H_4-MeO-TPCB, and M-sc_1, the vial was capped and placed in a 120 ºC oven for 20 h. After cooling to room temperature, the reaction suspension was then centrifuged at 6,000 rpm for 3 min to obtain ~4.5 mg of white solid. The solid was washed with fresh DMF (10 mL each time, 4x) and dispersed in DMF (1 mL). The prepared sc-PCN/DMF suspension was used for characterization and subsequent experimental procedures.

0.10 mL sc-PCN suspension was washed with MeOH (4 mL each time, 3x) and dispersed in 50:50 v/v ACN/EtOH (total volume 1 mL) for SEM, HAADF and STEM-EDS analysis.

Synthesis of cs-BBI

To a 20 mL Pyrex vial, $ZrCl_4$ (3.1 mg, 0.013 mmol), H_4-BBI (2 mg, 0.003 mmol), formic acid (3.5 mL),10 mL DMF, and Zr-BBI-Br_2/DMF suspension (0.1 mL, as prepared in previous section) were added. After sonication for 5 min to disperse seed crystals and dissolve $ZrCl_4$ and H_4-BBI, the vial was capped and placed in a 100 ºC oven for 20 h. After cooling to room temperature, the reaction suspension was then centrifuged at 6,000 rpm for 3 min to obtain ~5.7 mg of yellow solid. The solid was washed with fresh DMF (16 mL each time, 4x) and dispersed in DMF (1 mL). The prepared cs-BBI/DMF suspension was used for characterization.

0.10 mL cs-BBI/DMF suspension was washed with MeOH (4 mL each time, 3x) and dispersed in 50:50 v/v ACN/EtOH (total volume 1 mL) for SEM and STEM-EDS analysis.

Synthesis of ec-BBI

To a 20 mL Pyrex vial, $ZrCl_4$ (3.1 mg, 0.013 mmol), H_4-BBI (2 mg, 0.003 mmol), formic acid (3.2 mL), M-ec_2 (10 mg, 0.052 mmol), 10 mL DMF, and Zr-BBI-Br_2/DMF suspension (0.1 mL, as prepared in previous section) were added. After sonication for 5 min to disperse seed crystals and dissolve $ZrCl_4$, H_4-BBI, and M-ec_2, the vial was capped and placed in a 100 ºC oven for 20 h. After cooling to room temperature, the reaction suspension was then centrifuged at 6,000 rpm for 3 min to obtain ~5.4 mg of yellow solid. The solid was washed with fresh DMF (16 mL each time, 4x) and dispersed in DMF (1 mL). The prepared ec-BBI/DMF suspension was used for characterization.

0.10 mL ec-BBI/DMF suspension was washed with MeOH (4 mL each time, 3x) and dispersed in 50:50 v/v ACN/EtOH (total volume 1 mL) for SEM and STEM-EDS analysis.

Synthesis of sc-BBI

To a 20 mL Pyrex vial, $ZrCl_4$ (3.1 mg, 0.013 mmol), H_4-BBI (2 mg, 0.003 mmol), formic acid (3.2 mL), M-sc_2 (13 mg, 0.029 mmol), 10 mL DMF, and Zr-BBI-Br_2/DMF suspension (0.1 mL, as prepared in previous section) were added. After sonication for 5 min to disperse seed crystals and dissolve $ZrCl_4$, H_4-BBI, and M-sc_2, the vial was capped and placed in a 100 ºC oven for 20 h. After cooling to room temperature, the reaction suspension was then centrifuged at 6,000 rpm for 3 min to obtain ~5.4 mg of yellow solid. The solid was washed with fresh DMF (16 mL each time, 4x) and dispersed in DMF (1 mL). The prepared sc-BBI/DMF suspension was used for characterization.

0.10 mL sc-BBI/DMF suspension was washed with MeOH (4 mL each time, 3x) and dispersed in 50:50 v/v ACN/EtOH (total volume 1 mL) for SEM and STEM-EDS analysis.

¹H NMR of ec-PCN

About 5 mg of ec-PCN MOF, 1 mL DMSO-d_6 and 20 μL D_2SO_4 were added into a 1.5 mL centrifuge tube. The mixture was sonicated until all solid dissolved. The resulting solution was then transferred to an NMR tube and analyzed using a 500 MHz spectrometer. The NMR spectrum was then used to calculate the ratio of H_4-MeO-TPCB ligand and M-ec_1 modulator.

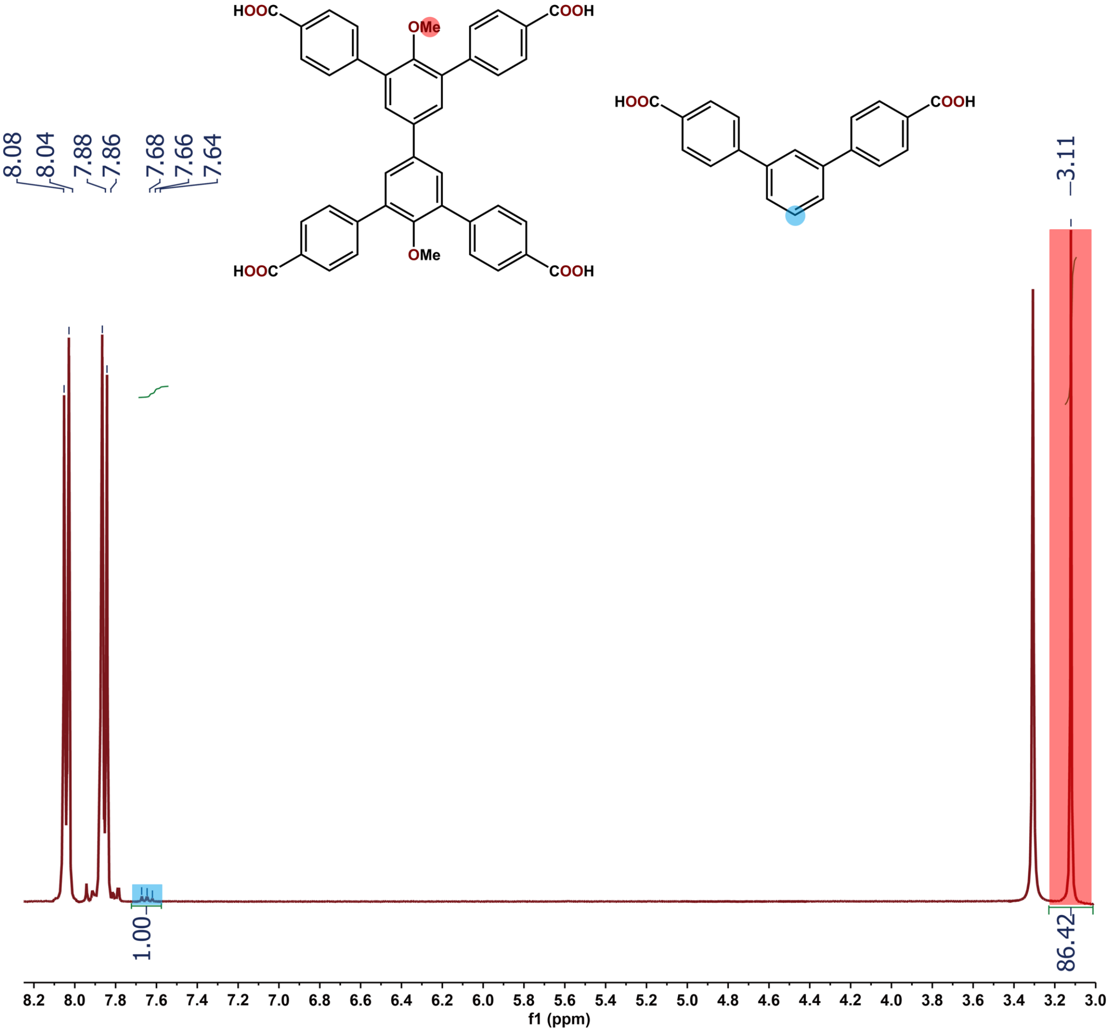

Figure S22. ¹H NMR spectrum of ec-PCN indicates a 0.07:1 ratio of M-ec_1 : H_4-MeO-TPCB.

PXRD Patterns of Binary Domain MOFs

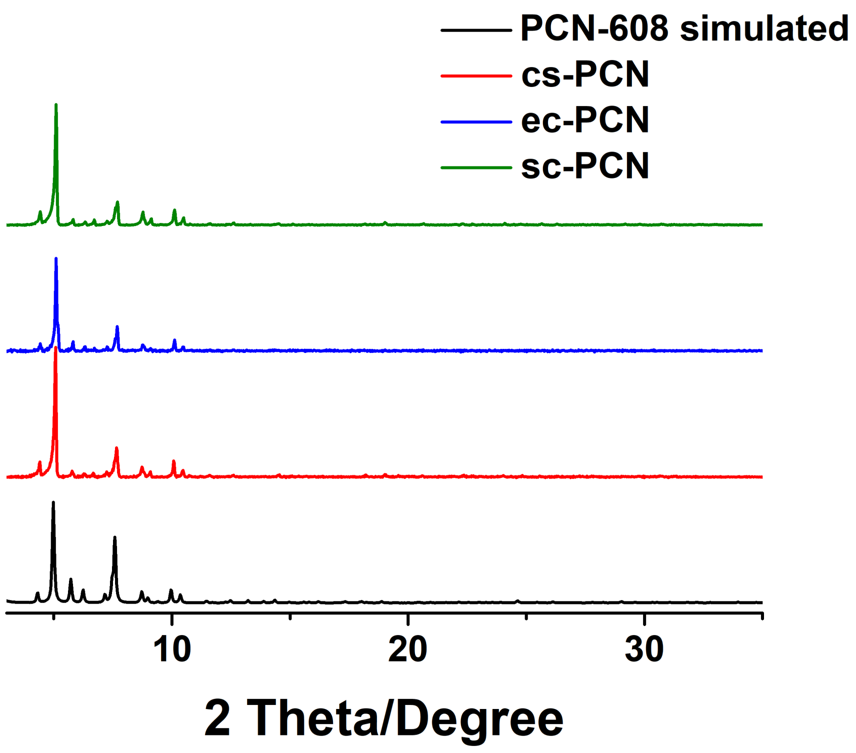

Figure S23. Simulated PXRD pattern of PCN-608 and experimental PXRD patterns of as-synthesized cs-PCN (red), ec-PCN (blue) and sc-PCN (green).

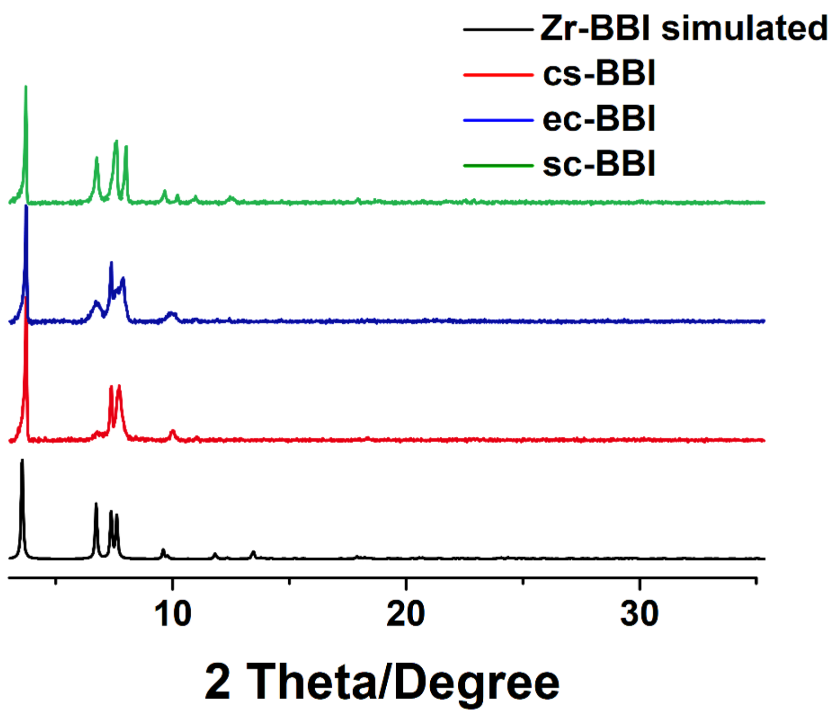

Figure S24. Simulated PXRD pattern of Zr-BBI and experimental PXRD patterns of as-synthesized cs-BBI (red), ec-BBI (blue) and sc-BBI (green).

Dimensions of Binary Domain MOFs

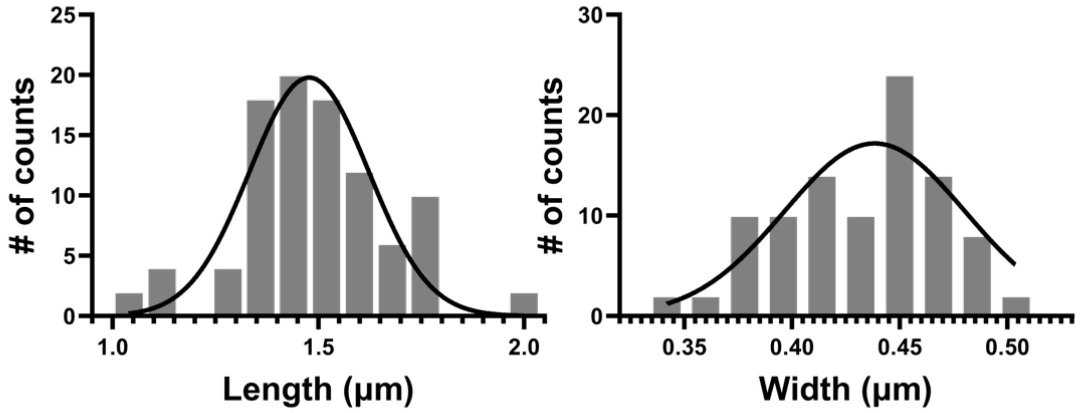

Figure S25. Length and width distributions of cs-PCN MOF crystallites fitted with Gaussian distribution curves (n=100 counts). The fit was performed using nonlinear regression.

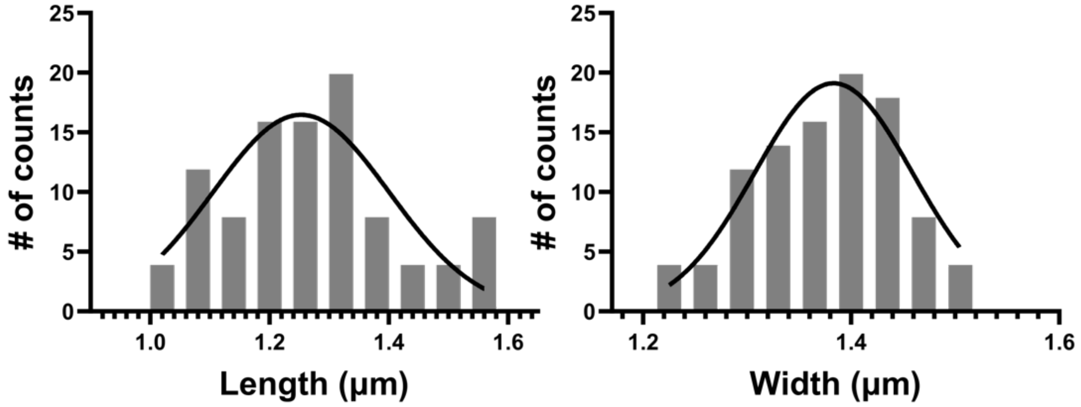

Figure S26. Length and width distributions of ec-PCN MOF crystallites fitted with Gaussian distribution curves (n=100 counts). The fit was performed using nonlinear regression.

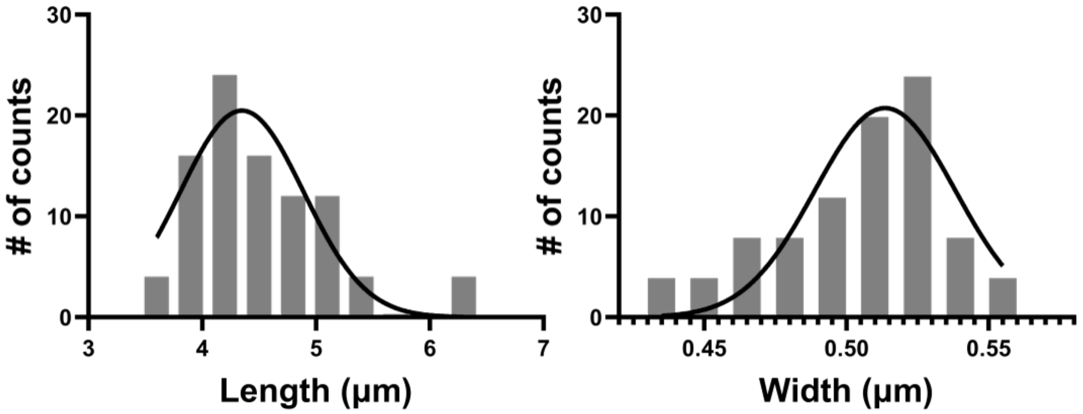

Figure S27. Length and width distributions of sc-PCN MOF crystallites fitted with Gaussian distribution curves (n=100 counts). The fit was performed using nonlinear regression.

Table S3. Average length, width, and aspect ratio of cs-PCN, ec-PCN, and sc-PCN MOFs (n=100 counts).

Binary MOFs	cs-PCN	ec-PCN	sc-PCN
**Length**	1.50±0.13 μm	1.27±0.11 μm	4.54±0.43 μm
**Width**	0.43±0.03 μm	1.38±0.06 μm	0.50±0.02 μm
**Aspect Ratio**	3.47±0.32	0.92±0.06	9.00±0.72

5. Syntheses and Characterization of Ternary Domain MOFs

Syntheses of Ternary Domain MOFs using cs-PCN Seeds

To a 20 mL Pyrex vial, $ZrCl_4$ (2 mg, 0.009 mmol), H_4-MeO-TPCB (2 mg, 0.003 mmol), trifluoroacetic acid (TFA, 0.14 mL), 10 mL DMF, and cs-PCN/DMF suspension (0.5 mL, as prepared in previous section) were added for normal ternary MOF growth. Either M-ec_1 (40 mg, 0.13 mmol) or M-sc_1 (60 mg, 0.15 mmol) was added to synthesize end-capped or side-capped ternary domain MOFs. After sonication for 5 min to disperse seed crystals and dissolve $ZrCl_4$, H_4-MeO-TPCB, and M-ec_1 or M-sc_1, the vial was capped and placed in a 120 ºC oven for 20 h. After cooling to room temperature, the reaction suspension was then centrifuged at 6,000 rpm for 3 min to obtain ~3.5 mg of white solid. The solid was washed with fresh DMF (10 mL each time, 4x) and dispersed in DMF (1 mL). 0.10 mL MOF/DMF suspension was washed with MeOH (4 mL each time, 3x) and dispersed in 50:50 v/v ACN/EtOH (total volume 1 mL) for SEM, HAADF, and STEM-EDS analysis.

Syntheses of Ternary Domain MOFs using ec-PCN Seeds

To a 20 mL Pyrex vial, $ZrCl_4$ (2 mg, 0.009 mmol), H_4-MeO-TPCB (2 mg, 0.003 mmol), trifluoroacetic acid (TFA, 0.14 mL), 10 mL DMF, and ec-PCN/DMF suspension (0.5 mL, as prepared in previous section) were added for normal ternary MOF growth. Either M-ec_1 (40 mg, 0.13 mmol) or M-sc_1 (60 mg, 0.15 mmol) was added to synthesize end-capped or side-capped ternary domain MOFs. After sonication for 5 min to disperse seed crystals and dissolve $ZrCl_4$, H_4-MeO-TPCB, and M-ec_1 or M-sc_1, the vial was capped and placed in a 120 ºC oven for 20 h. After cooling to room temperature, the reaction suspension was then centrifuged at 6,000 rpm for 3 min to obtain ~3.5 mg of white solid. The solid was washed with fresh DMF (10 mL each time, 4x) and dispersed in DMF (1 mL). 0.10 mL MOF/DMF suspension was washed with MeOH (4 mL each time, 3x) and dispersed in 50:50 v/v ACN/EtOH (total volume 1 mL) for SEM, HAADF, and STEM-EDS analysis.

Syntheses of Ternary Domain MOFs using sc-PCN Seeds

To a 20 mL Pyrex vial, $ZrCl_4$ (2 mg, 0.009 mmol), H_4-MeO-TPCB (2 mg, 0.003 mmol), trifluoroacetic acid (TFA, 0.14 mL), 10 mL DMF, and sc-PCN/DMF suspension (0.5 mL, as prepared in previous section) were added for normal ternary MOF growth. Either M-ec_1 (40 mg, 0.13 mmol) or M-sc_1 (60 mg, 0.15 mmol) was added to synthesize end-capped or side-capped ternary domain MOFs. After sonication for 5 min to disperse seed crystals and dissolve $ZrCl_4$, H_4-MeO-TPCB, and M-ec_1 or M-sc_1, the vial was capped and placed in a 120 ºC oven for 20 h. After cooling to room temperature, the reaction suspension was then centrifuged at 6,000 rpm for 3 min to obtain a white solid. The solid was washed with fresh DMF (10 mL each time, 4x) and dispersed in DMF (1 mL). 0.10 mL MOF/DMF suspension was washed with MeOH (4 mL each time, 3x) and dispersed in 50:50 v/v ACN/EtOH (total volume 1 mL) for SEM, HAADF, and STEM-EDS analysis.

PXRD Patterns of Ternary Domain MOFs

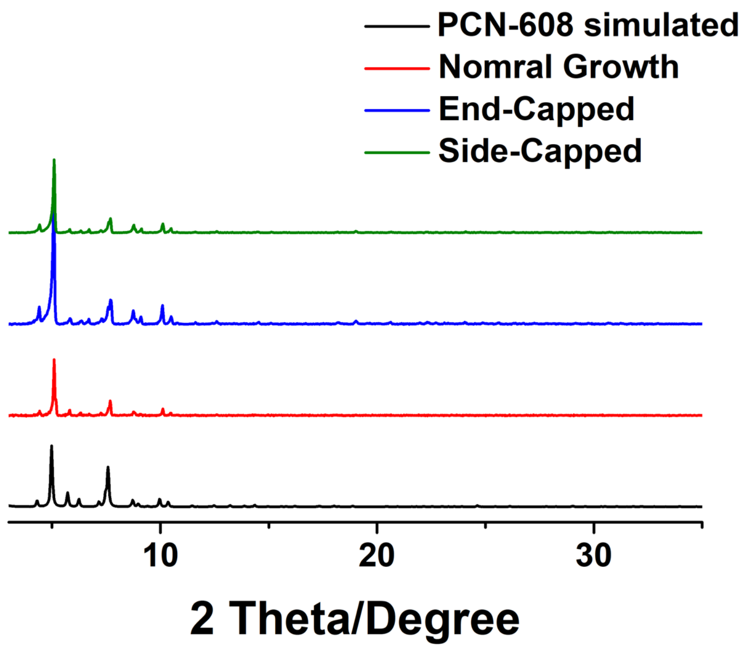

Figure S28. Simulated PXRD pattern of PCN-608, and experimental PXRD patterns of as-synthesized normally grown (red), end-capped (blue) and side-capped (green) ternary domain PCN MOFs using cs-PCN seeds.

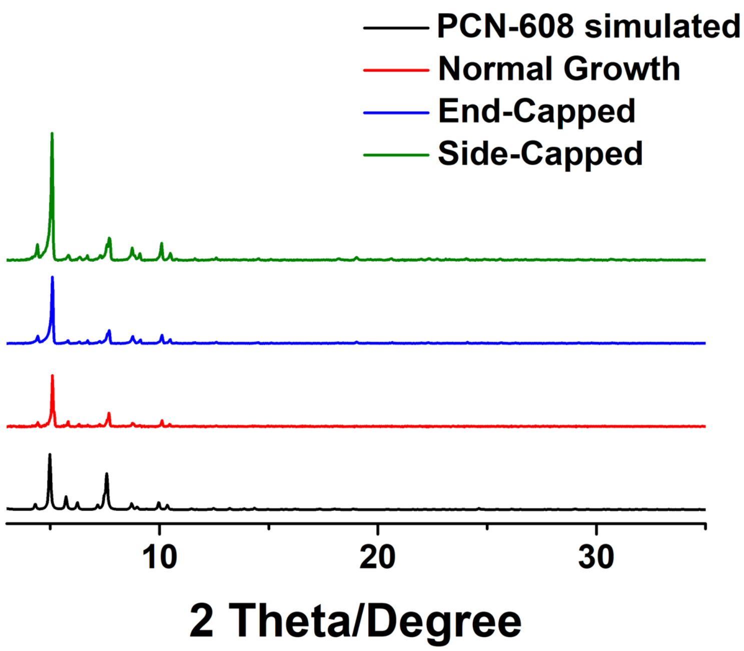

Figure S29. Simulated PXRD pattern of PCN-608, and experimental PXRD patterns of as-synthesized normally grown (red), end-capped (blue) and side-capped (green) ternary domain PCN MOFs using ec-PCN seeds.

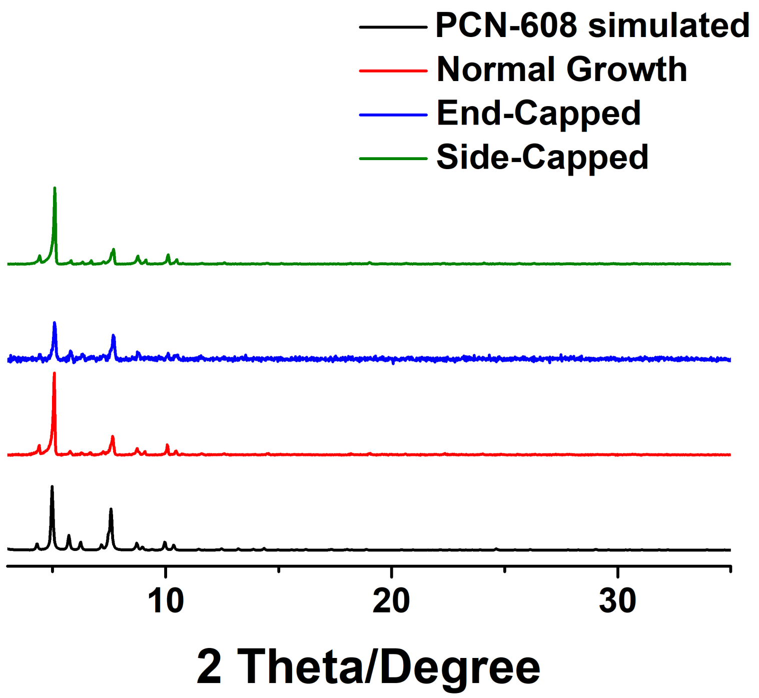

Figure S30. Simulated PXRD pattern of PCN-608, and experimental PXRD patterns of as-synthesized normally grown (red), end-capped (blue) and side-capped (green) ternary domain PCN MOFs using sc-PCN seeds.

6. References

(1) C. A. Schneider, W. S. Rasband, K. W. Eliceiri, *Nat. Methods* **2012,** *9*, 671–675.

(2) J. Schindelin, I. Arganda-Carreras, E. Frise, V. Kaynig, M. Longair, T. Pietzsch, S. Preibisch, C. Rueden, S. Saalfeld, B. Schmid, J. Y. Tinevez, D. J. White, V. Hartenstein, K. Eliceiri, P. Tomancak, A. Cardona, *Nat. Methods* **2012,** *9*, 676–682.

(3) J. Pang, S. Yuan, J. Qin, C. Liu, C. Lollar, M. Wu, D. Yuan, H.-C. Zhou, M. Hong, *J. Am. Chem. Soc.* **2017,** *139*, 16939-16945, DOI: 10.1021/jacs.7b09973.

(4) M. Xu, P. Cai, S. Meng, Y. Yang, D. Zheng, Q. Zhang, L. Gu, H.-C. Zhou, Z.-Y. Gu, *Angew. Chem. Int. Ed.* **2022,** *61*, e202207786.

(5) Z. Shi, L. Qin, H. Zheng, *Dalton Trans.* **2017,** *46***,** 4589-4594.

(6) X. Wang, Y. Zhang, Z. Shi, T. Lu, W. Wang, B. Li, *ACS Appl. Mater. Interfaces* **2021,** *13* (45), 54217-54226.

(7) Y. Kumar, Y. Jaiswal, A. Kumar, *Eur. J. Org. Chem.* **2018,** 494–505.
